# Designing the Ethylene Factory for Products of Carbon Dioxide Reduction: Techno-Economic and Life Cycle Assessments

**DOI:** 10.1021/acssuschemeng.4c10485

**Published:** 2025-09-11

**Authors:** Ariane Silveira Sbrice Pinto, Nalan Gulpinar, Fang Liu, Elizabeth A. Gibson, Linsey Fuller, Philip Souter

**Affiliations:** † Business School, Management Department, 3057 Durham University, Durham DH1 3LB, England, United Kingdom; ‡ Energy Materials Laboratory, Chemistry, School of Natural and Environmental Science, Newcastle University, Newcastle-upon-Tyne NE1 7RU, England, United Kingdom; § Procter and Gamble, 8552 Newcastle Innovation Centre, Whitley Road, Longbenton, Newcastle upon Tyne NE12 9TS, United Kingdom

**Keywords:** ethene, supply chain design, green credits, carbon capture and utilization, uncertainty analysis

## Abstract

The global ethylene market is rapidly expanding, and as demand grows, emissions are projected to rise, underscoring the urgent need for sustainable technologies to mitigate its carbon footprint. An original manufacturing approach integrated carbon capture and utilization (CCU), esterification, and dehydration to explore the utilization of intermediate chemicals for a circular economy. Initially, CO_2_ was reduced to formic acid via electrocatalysis. Subsequently, esterification with ethanol produced ethyl formate, which was thermally catalyzed into ethylene. Comprehensive techno-economic and life cycle assessments identified opportunities and bottlenecks in designing this novel supply chain. Despite high production costs ($4.79 ± 1.19/kg), the environmental performance was promising. The LCA indicated a low carbon footprint, with up to 86% of emissions falling below benchmark levels (average 0.88 ± 0.55 kg CO_2_ eq/kg), whereas other burdens exhibited an inverse trend. An original framework combining TEA-LCA, sensitivity analysis (SA), and uncertainty analysis (UA) was applied to forecast variability effects on the net present value (NPV) and product carbon footprint (PCF). Wastewater treatment, auxiliary materials, and CCU were primary contributors to the PCF’s uncertainty, leading to up to 90%, 45%, and 35% of the total variance, respectively. Operational expenditures (OpEx) related to power and raw materials accounted for up to 90% of NPV uncertainty. In contrast, total capital investment (TCI) and revenue (product and green credits from emissions-trading schemes, ETS) together contributed less than 10%. Improvements in yield, optimization of downstream processes, economic incentives, and/or the creation of a market for industrial flue gases as extra revenues are still necessary to compensate for high production costs and enable the deployment of the proposed technology to mitigate global warming burdens from ethylene production. In a complex decision-making process, technology mapping, cutoffs to fit the readiness level, green certification identification, UA, and SA for a combined TEA -LCA were identified as essential steps to guide future developments.

## Graphical abstract

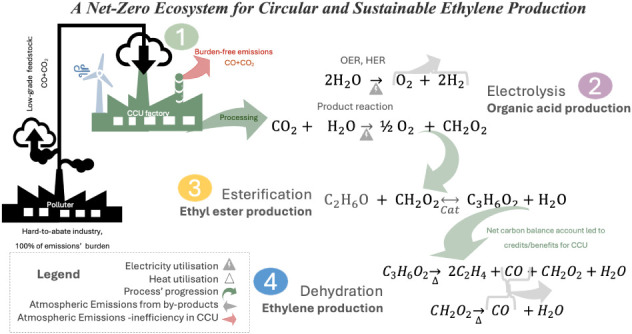

## 1 Introduction

Ethylene production has grown substantially due to rising demand in pharmaceuticals, agriculture, and polymer manufacturing.^1^ In 2022, global ethylene production reached 225 million metric tons,^2^ emitting ∼342 million MT of CO_2eq_. Emissions are forecast to rise by 17 million MT of CO_2eq_ between 2024 and 2033, assuming 5.72%^1^ market growth and continued reliance on hydrocarbon cracking.^3^ In this context, sustainable technologies are urgently needed to decarbonize ethylene’s carbon footprint. Replacing conventional carbon sources (e.g., naphtha, ethane, and coal) in ethylene’s supply chain with eco-friendly alternatives can promote a circular economy while moving toward the 2050 net-zero ambition. Carbon capture and utilization (CCU) emerges as a promising strategy to mitigate ethylene’s carbon footprint.^4,5^

CCU captures CO_2_ of industrial flue gases for use as a raw material in chemical’s production.^5^ The advantages include the reduction of emissions from ethylene and its products, capturing emissions from hard-to-abate industries (e.g., CO, CO_2_) by replacing petrochemical feedstocks, and mitigating the global warming effect. Although producing ethylene from CCU could be promising, challenges have been identified. Economic barriers include high production costs due to low yields and excessive energy demand, which could result in a product that is approximately 3-fold more expensive than forecast market prices.^6^ Economic analysis must compare unit costs against market prices, while the complexity of environmental analysis also needs a baseline to certify benefits for emission trading schemes (ETS) and prevent double counting.^7–9^ To mitigate ethylene’s carbon footprint via CCU, green upstream technologies are still required, mainly to provide renewable energy.^6^ Indeed, opportunities and bottlenecks depend on the technology and require a full TEA-LCA study, including uncertainties and sensitivity analyses.^9^

Ethylene can be produced from CO_2_ either directly^10^ or via intermediates.^11,12^ Despite involving extra processing, an intermediate route encourages cross-sector synergies^8^ to support investments in low technology readiness level (TRL) technologies, such as CCU factories. As a versatile intermediate, formate/formic acid is a particularly relevant intermediate since its production cost is the lowest among other CO_2_-derived products—up to 50% cheaper than CCU ethylene.^13^ As a critical building block, formate/formic acid has wide applications,^14^ which stimulate investment and foster the deployment of CCU. Formic acid can be produced with bio/electro-catalysis.^8,15^ The productivity of formic acid could reach 1.2 M in 50 h^8,15^ (TRL ∼4)^16^ by using gaseous CO_2_ and sodium bicarbonate as catholyte.^14,16,18^ Pilot tests to convert up to 146 kg of CO_2_ per day into 110 kg of formate/formic acid per day^17^ demonstrated process feasibility^18^ – exciding 80% of faradaic efficiency, active area of 40,000 cm^2^, and up to 1 A cm^‑2^.^15,18^

Beyond the technical feasibility, CCU aligns with sustainable development goals^19^ by mitigating up to 80%^20^ of PCF and enabling the supply chain design for carbon-neutral/negative products.^8^ Recovering organic acid into higher-value products, such as ethylene, is promising, as CO_2_-to-formate/formic acid production requires 30% less electricity cost per product unit than direct electroreduction of CO_2_-to-ethylene.^13,21^ Key economic challenges for CO_2_ electroreduction include energy and feedstock costs, with target prices at $0.07/kWh and $40/ton of CO_2_,^22,23^ respectively. Energy prices impact at least 40% of ethylene’s unit cost^13,21^ manufactured with electrocatalysis, reinforcing the potential benefits of utilizing intermediates instead.^27–29^ Product grade and regional economics will also drive cost variability for CCU factories, ranging from $710 ^8^ to $2,095/ton.^23–25^

Similarly to the ethanol dehydration,^25–27^ producing ethylene from ethyl esters faces byproduct challenges.^28^ Although the production of light olefins from esters remains not fully explored, the direct dehydration of ethanol toward ethylene shows that breakeven is possible using low-grade feedstock, as impurities did not affect ethylene quality.^32^ Breakeven for ethylene from ethanol was achieved at $455/ton for ethanol and $1,000/ton for ethylene.^41^ Plant design bottlenecks stemmed from high power consumption during ethanol dehydration and purification, recovering the carbon from the feedstock in ethylene (85%*wt)*, ethanol (12%*wt*), and higher olefins (2%*wt*).^26^ Ethylene’s carbon footprint via ethanol dehydration ranged from −2.10 to 13.97 kg CO_2eq_ /kg ethylene, varying if allocation benefits for biogenic CO_2_ storage in bioplastics products were discounted or not.^29^ Fertilizer use drove 46% of emissions in the sugar beet biorefineries context,^29^ suggesting that CO_2_ as a carbon source could substantially cut ethylene’s footprint without relying on credits due to a specific application. Besides, the esterification of formic acid and ethanol to produce ethyl formate^30^ is promising, since ethyl formate^30–33^ is an intermediate chemical that can be either sold as a CCU product or as set here, used as a precursor of ethylene.^34^

Briefly, integrating CO_2_ electroreduction, esterification, and dehydration could be advantageous in comparison to the ethanol dehydration due to the potential of reducing the impact of ethanol on ethylene’s PCF while alternatively allowing the valorization of aqueous products (a mixture of organic acids and alcohols^35^) into ethylene or ester products,^36,37^ with minimal purification. The esterification reaction^32,38^ occurs in the presence of concentrated sulfuric acid,^39^ followed by extractive distillation with ethylene glycol^40^ (TRL ∼8–9). Then, ethyl ester^34^ decomposes to ethylene at high temperatures with a zeolite catalyst. Despite the opportunities of combining these technologies to produce ethylene, this process synthesis has not yet been explored to identify hotspots in industrial manufacturing.

Here, a comprehensive techno-economic and environmental discussion for identifying opportunities and bottlenecks was provided. The technicalities of the ETS were critically examined with an original framework to fully combine TEA-LCA, global sensitivity, and uncertainty analyses for evaluating CCU technologies.

## 2 Methods

### 2.1 Inventory

The ethylene factory integrates electrocatalysis, esterification, and dehydration reactions. CO_2_ is reduced to formic acid via electrocatalysis. Esterification with ethanol yields ethyl formate, which subsequently decomposes to ethylene. The primary feedstocks for esterification might be CO_2_ from flue gases and ethanol from either renewable sources or CCU. Among potential CO_2_ feedstocks, blast furnace gas (BFG) from steel production is promising in the context of meeting the decarbonization targets of the UK’s industrial clusters due to high emissions (∼84.5 million tonnes/year).^41,42^ Typical steel production (9,000 tonnes of steel/day^43^ generates ∼2.50 tonnes of BFG/tonne of steel, equating to ∼900 tonnes of BFG/h for CCU factories. The consumption of 100 tonnes/h BFG was set to produce ∼30,177 tonnes of ethylene/annum. BFG has the highest emissions among thermal nonrenewable fuels^44^ usually with a CO_2_ concentration of ∼23 mol %, as detailed in Table S1.

Formic acid was produced from CO_2_ in an electrocatalytic cell,^15^ as described by Pinto et al.^8^ High and low productivity were considered during the electrolysis: HP (ideal forecast) and LP (experimental data), respectively. The productivity of formate/formic acid in the electrolyzer reached ∼0.024M/h 1 ^15^ for LP scenario, which led to ∼30% of the theoretical yield (CO_2_H_2_/CO_2_ ≅1.046*wt*). HP was set as ∼75% of the theoretical yield by fixing other conditions equal to LP. A maximum of 5%*wt* of bicarbonate in the catholyte was set to mitigate salt precipitation.^45,46^ The summary of LP operational conditions for the electrolyzer is displayed in Table 1 (TRL = 4).^16^ Electrodialysis was used to separate formic acid from formate^47^ and to concentrate the acid up to 3-fold.^48^ Liquid–liquid (L–L) extraction before distillation was essential for the formic acid purification.^49^ The formic acid solution was concentrated prior to the distillation process using methyltetrahydrofuran (MeTHF), which was recovered in the stripping column and recycled.

**Table 1:** ElectrolyzerOperational Condition of the electrolyzer.^15^

Parameter	Experimental values
Formate/formic acid yield	1.2 M (in 50 h)
Productivity^a^	∼0.024 M/h
FE	81%
*Main auxiliary materials*	*(NaOH, Na*_2_ *CO*_3_, *Glycerol)*
Catholyte	0.1 M NaHCO_3_ /Na_2_CO_3_
Anolyte	3 M NaOH; 0.10 M glycerol

a Estimated with experimental data.

Formic acid reacted with ethanol in a continuous stirred tank reactor (CSTR) to produce ethyl formate^30,31^ -the direct precursor of ethylene. The esterification reaction^32,38^ proceeded with a sulfuric acid catalyst, followed by extractive distillation with ethylene glycol,^40^ which was subsequently recycled. The ethanol production was not modeled, and the inventory of its production from renewable sources is available at Ecoinvent database, as described in Section 2.2.

Ethylene (99%*wt-* main product, MP) from ethyl esters^38^ was manufactured via dehydration at 250° (zeolite catalyst) in packed bed reactors (PBRs) with 98% selectivity.^34^ CO, CO_2_H_2_, and H_2_O were coproducts. Figure 1 displays the block diagram, and the process diagram used for modeling is available as (Figure S1). The mass balance, overall reactions, yields, and carbon accounting for each system illustrated in Figure 1 are provided in Table 2. Auxiliary materials demand, power, and utilities usage are listed in Table S2.

**Table 2:** Overall Reactions, Yields, and Carbon Accounting^c^

In-/out-puts	[lower bound, LP,			Upper bound, HP]			Stoichiometry		Product: CCU on site^a^ [wt %]		
Gas[kg/h]	IN	OUT	NET/LOSS	IN	OUT	NET/LOSS	Overall reaction	Yield [wt]	LP	HP	Carbon-track[mol %] and keynotes
BFG	1 × 10^5^	7 × 10^3^	1 × 10^5^	1 × 10^5^	3 × 10^4^	9 × 10^4^	-				Zero burdens^52^
CO_2_	3 × 10^4^	3 × 10^4^	7 × 10^3^	4 × 10^4^	1 × 10^4^	3 × 10^4^	Na_2_CO_3_ + CO_2_ + H_2_O → 2NAHCO_3_ (20wt%,LP) <χ40^b^ wt %,HP)	-CO_2‑‑locked_	22*	71**	100% from BFG’s CO_2_
CO	1 × 10^4^	1 × 10^4^	0	1 × 10^4^	1 × 10^4^	0	-				Although CO shall be potential consumed, its consumption was neglected due to data limitation, and it was 100% part of air emissions.
Impurities	6 × 10^4^	6 × 10^4^	0	7 × 10^4^	7 × 10^4^	7 × 10^4^	-				nonorganic (H_2_; N_2_)
**Intermediates**[kg/h]											
HCO_2_H	0	8 × 10^3^	1 × 10^3^	0	3 × 10^4^	2 × 10^3^	$$\begin{array}{l} {\mathrm{CO}}_{2}{\mathrm{+H}}_{2}O\to {\mathrm{1/2O}}_{2}{\mathrm{+CH}}_{2}O_{2}\\ \left(\mathrm{30 wt \% , LP}\right)\mathrm{<\chi <75 wt \%,HP)} \end{array}$$	1.05 CH_4_O_2_: CO_2_	28	103	CO_2_ (single pass) was 100% from BFG and only the CCU used to produce ${\mathrm{CH}}_{2}O_{2}$ was offset from ethylene’s PCF.
C_2_H_5_OH (96 wt %)	7 × 10^3^	4 × 10^2^	6 × 10^3^	2 × 10^4^	1 × 10^3^	2 × 10^4^	CO2H2+C2H6O→C3H6O2+H2O	1.61 C_3_H_6_O_2_: CO_2_	149	144	Carbon was 67% from alcohol and 33% from organic acid to generate 1 molecule of ester. Ethanol’s embodied carbon was estimated with renewable feedstocks processing (max. burden from the supply chain).
C_3_H_6_O_2_	0	1 × 10^4^	3 × 10^2^	0	4 × 10^4^	1 × 10^3^					
**Product**[kg/h]											
C_2_H_4_	0	3 × 10^3^	0	3 × 10^3^	0	1.E+4	$$\begin{array}{l} {\mathrm{2C}}_{3}H_{6}O_{2}\to {2C}_{2}H_{4}{\mathrm{+CO+CH}}_{2}O_{2}+H_{2}O\\ \left(\mathrm{\chi ∼97 wt \%}\right) \end{array}$$	0.38 C_2_H_4_: C_3_H_6_O_2_	47	51	67% of total carbon was allocated to C_2_H_4_ and, at least, 33% allocated to CO and CH_2_O_2_ combined.
CO byproducts	0	1 × 10^3^	00	1 × 10^3^	0	2.E+4		0.19 CO: C_3_H_6_O_2_	18	25	
CO byproducts/impurities	0				0		$$\begin{array}{l} {\mathrm{CH}}_{2}O_{2}\to {\mathrm{CO+H}}_{2}O\\ \left(\mathrm{\chi =100 wt \%}\right) \end{array}$$	0.61 CO: CH_2_O_2_	0	56	All CH_2_O_2_ molecules generated 100% of CO. CO environmental burden was equivalent to CO_2_.

a The ratio between the mass of the specified product and CO_2_ BFG-NET. , except for * and **, which were calculated by NET/ IN CO2 ratio.

b Mass conversion and convergence/model errors varied from 10 to 20%.

c Mass conversion and convergence/model errors varied from 10 to 20%.

The scope was to identify hotspots, opportunities, and future challenges of designing the supply chain using the former process synthesis and to evaluate the utilization of BFG by considering operational conditions from experimental assessments. Optimization was out of scope due to limited data availability for this new approach.

### 2.2 Life Cycle Assessment

The functional unit (FU) for all scenarios was 1 kg of MP. Impacts were assessed using the Environmental Footprint (EF, v3.0) with 24 characterization factors.^50^ Byproducts were not considered in the assessment. The global warming potential (GWP100a) was used to forecast the PCF both with (PCF_Net_) and without carbon offsetting (PCF_nonoffset_). The offset benefits evaluated the potential mitigation of the emissions due to captured CO_2_ and the treatment of aqueous waste. Avoided emissions were calculated only when the PCF was lower than the benchmark emissions from the hydrocarbon cracking process; otherwise, green credits (CBios) were not included.^7^ Similar to waste valorization into high-value products,^42,51^ the “polluter-pays” principal^52^ was applied for the BFG, which was a burden-free stream in the CCU factory. The estimated indirect GWP100a for CO considered CH_4_ feedback^53^ only, at 1.0 kg CO_2eq_ /kg CO. The evaluation of different time horizons^53^ for GWP was outside the scope of this work. The system boundary for the cradle-to-gate approach was illustrated in Figure 1. The list of background processes was provided in Table S5.

### 2.3 Techno-Economic Analysis

The net present value (NPV), revenue, operational expenditures (OpEx), capital expenditures of equipment (CapEx^E^), and total capital investment (TCI) were used to compare the technologies’ feasibility. SuperPro Design software was used to estimate other costs, except for the electrolyzer’s capital investment that was forecasted based on the electricity demand of the PEM system.^54^ The analysis was conducted for the year 2024, with all financial figures presented in USD. The construction period spanned 30 months, followed by a startup phase of 4 months. The project’s lifespan was projected to be 30 years, with a default internal rate of return of 7% and an inflation rate of 4%. The OpEx for the project included costs for materials, labor-dependent entries, facility-related expenses, consumables, laboratory analysis, utilities, and miscellaneous expenditures. The exchange rates for 2019 and 2024 were 0.784 GBP:1 USD and 0.810 GBP:1 USD, respectively. The market prices for each intermediate chemical, utilities, electricity, and auxiliary materials are summarized in the SM.

### 2.4 Sensitivity and Uncertainty Analyses

To identify the sources of uncertainties in the foreground processes, two operational conditions were selected: default and ideal scenarios. Default conditions used the experimental data provided in Table 1,^15^ representing the LP scenario. A future ideal scenario assumed a potential increase in the TRL of the CCU process, which delivered higher productivity (HP) due to the higher availability of CO_2_. This productivity range (from LP to HP) was used to forecast uncertainties for the factory’s emissions.

Additional revenue streams in the ethylene factory were also considered as a potential source of uncertainty. Two approaches were evaluated: creating a market for industrial flue gases (by pricing CO_2_ captured by the technology) and implementing carbon trading schemes (generating credits from carbon offsetting). Uncertainties included potential charges associated with treating BFG, with prices estimated based on the technology’s CO_2_ capture capacity and market value. Economic credits from the ETS were included only if the PCF of the CCU factory was lower than benchmark emissions. To forecast the source of uncertainties on the foreground processes, the standard deviation (STD) of the two operational conditions was considered (LP and HP scenarios). Table 3 shows the average value and STD of the economic parameters and productivity.

**Table 3:** Variability to Forecast Potential Uncertainties of CCU Factory Deployment

Variable [unit]	Average	STD
NPV [$] (7.0% Interest)	–2.2 × 10^09^	1.1 × 10^09^
TCI [$]	3.3 × 10^08^	2.0 × 10^08^
OpEx [$/y]	2.5 × 10^08^	1.4 × 10^08^
CapEx^E^[$]	5.0 × 10^07^	3.0 × 10^07^
Revenue [$/y]	4.6 × 10^07^	3.5 × 10^07^
Unit production cost [$/kg]	4.79	1.19
Ethylene productivity [kg/y]	5.8 × 10^07^	4.4 × 10^07^

Extra revenues for the ethylene factory were considered in the NPV calculation. Economic credits from the ETS were only included if the PCF of the CCU factory was lower than the benchmark emissions. Since the carbon trading market can be volatile, the green credit price could change from 50 to 150 USD/ton. of CO_2eq_ .^55–57^ The default price for carbon trading was set at 100 USD/ton of CO_2eq_ with a deviation of ±50%. The carbon offsetting was calculated as the difference in PCF when producing ethylene via CCU versus naphtha cracking technologies (PCF_Net_ < PCF_Benchmark_). The mass and energy balance (M and EB) proportionalities were preserved by considering logarithmic adjustments among the average, and lower-upper bound values (constrained by the scenarios limits).

The geometric standard deviation and Ecoinvent’s pedigree matrix for data quality analysis were used to set the deviation range for the background processes.^58^ Random sampling was conducted within the range of variability to perform the global density analysis and evaluate the uncertainty of the outputs (NPV and PCF).^8^ The deviation range of costs was set at 40% of the default value (average value between LP and HP scenarios). The summary of default values and the deviation range is available in Tables S2 and S6.

The UK’s tax scenario was considered for the uncertainty analysis.^51^ The Sobol sampler was used in global sensitivity analysis^59^ and to generate enough random points to access the uncertainty (1 × 10^+8^ points).^9^ It is important to note that different methods can be used to foresee uncertainties,^60^ but the selected approach provided enough resolution to identify hotspots in the economic and environmental aspects of the analysis.

### 2.5 Software and Database

SuperPro Process Design software (TEA and foreground inventory), Brightway2 (LCA software),^61^ and Microsoft Visual Studio Code (Python interface) were used for data acquisition and curation. The cutoff database from Ecoinvent was used for background inventories.^3^ Indicators from the Environmental Footprint (EF, v3.0) and global normalization index were used to display LCIA.^50^

## 3 Results and Discussion

### 3.1 Life Cycle Assessment

Among LCA impact factors, the most critical for industrial decarbonization is the PCF (or GWP100a). Ethylene production via the selected pathway resulted in an average PCF of 0.98 kg CO_2eq_ /FU, representing a 36% reduction compared to the benchmark. Contribution analysis and additional characterization factors identified bottlenecks to achieving carbon-neutral/negative emissions, as presented in Figure 1a,b, respectively.

Approximately 22–71 wt % of CO_2_ from BFG was locked as carbonate (Table 2). The minimal electrocatalytic conversion of CO_2_ into CH_2_O_2_ was 30%*wt* (LP) (Figure 1), resulting in 28 wt % utilization of CO_2_ from the BFG, as demonstrated in the carbon accounting from Table 2. The CCU reduced 15–40% of the total PCF_nonoffset_ through avoided emissions credits, as shown in Figure 2a. Analogously to CCU, wastewater disposal led to environmental benefits. These combined strategies achieved carbon-neutral emissions for the LP scenario (0.33 kg of CO_2eq_ /FU), while the PCF for the HP scenario decreased by 43% (1.63 kg of CO_2eq_ /FU). Anaerobic digestion of aqueous waste reduced PCF_nonoffset_ by 8% (HP) and 36% (LP). Notably, while increased recycling and reduced wastewater in the HP factory lowered raw material utilization impacts, the overall PCF was offset by effluent treatment credits. This dynamic benefited the LP scenario, where its higher offsetting of wastewater treatment emissions led to near-carbon-neutral performance, unlike the HP scenario.

**Figure 1:**
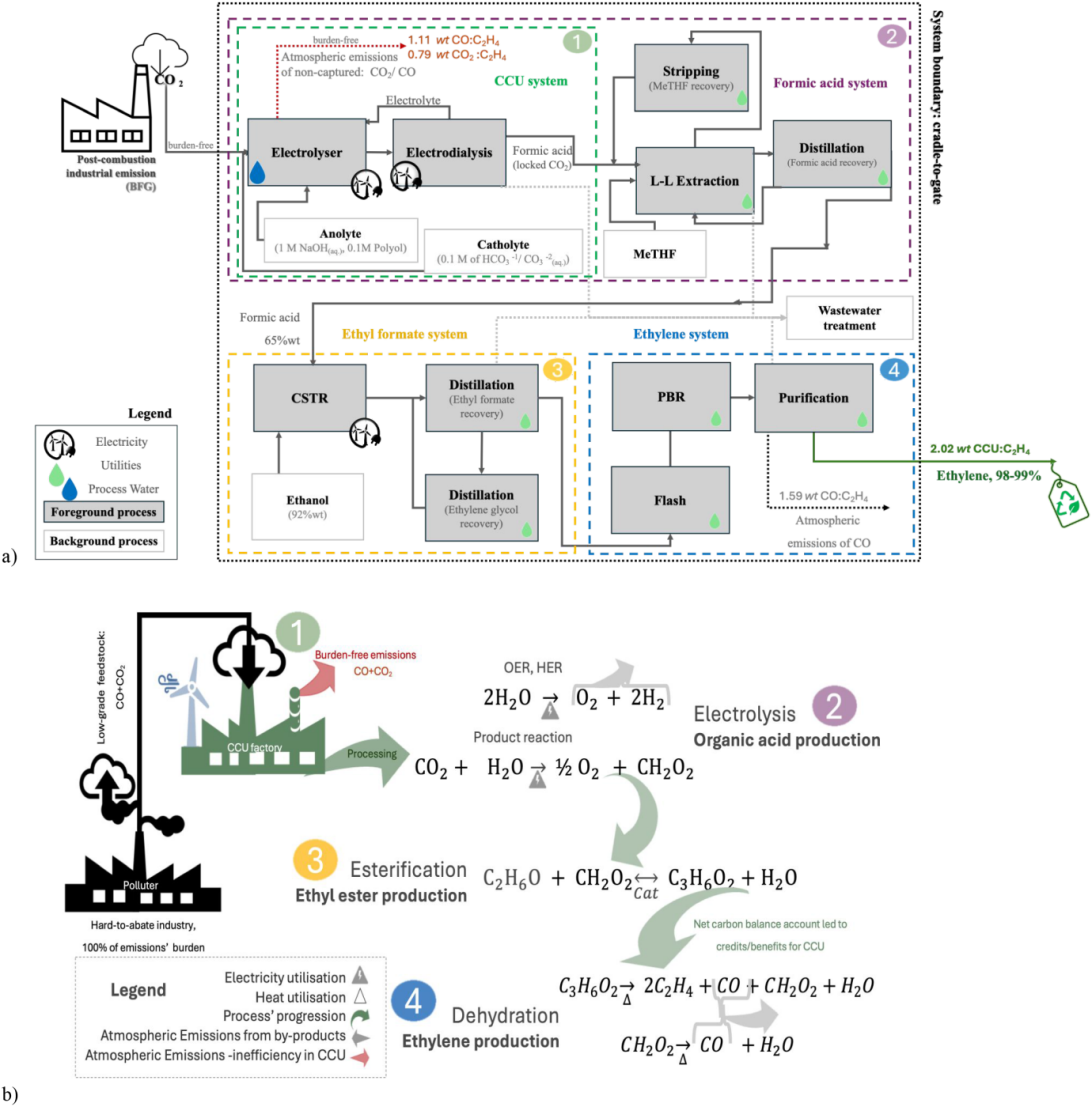
Process design. Block diagram of ethylene production (a). Overview of intermediate utilization, indicating carbon sources and reactions (b). The foreground process was modeled with estimates described in Section S1.1, while the background process used ecoinvent inventories, as listed Section S1.3. The complete breakdown of each reaction set is available eqs (S2–S8).

**Figure 2:**
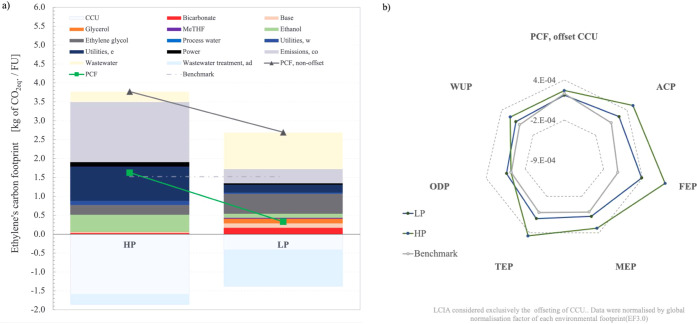
Environmental analysis. Product carbon footprint (PCF) breakdown per input (a). LCA (b).

The selection of background technology within the supply chain is critical to the overall PCF. Although ethanol was identified as a major contributor to the PCF at high productivity (HP), in a circular economy context, its carbon footprint may be reduced. Coproduction of ethanol from CO_2_ and biobased carbon sources has been evaluated, leveraging CO_2_-rich streams from fermentation processes through CCU.^62^ Biorefineries operating with both corn and CO_2_ as carbon sources have been proposed to achieve an ethanol footprint near carbon neutrality.^63^ However, in the European context, sugar beet was identified as a more viable feedstock to reduce emissions in ethylene manufacturing.^64^ The selection of this technology to infer the impact of ethanol provided its maximum contribution to the system since other emerging technologies with market potential should be as good as the market as usual or provide carbon offsets, resulting in additional benefits for the overall supply chain. In the supply chain context, carbon accounting remains challenging for circular economy systems, mainly due to issues related to benefits/burdens allocation.^9,65^

Allocation of environmental credits from CO_2_ use must be carefully conducted to avoid double counting.^68^ In circular systems utilizing BFG, burdens/benefits must be properly allocated between the supplier (polluter) and the buyer (CCU factory), as recommended for specific European sectors.^66^ Given that life cycle assessment (LCA) modeling philosophy was beyond this study’s scope, the nonpure flue gas was treated as a zero-burden stream, assigning emission penalties to the polluter and credit for CO_2_ utilization solely to ethylene. Allocation in carbon accounting will be especially important for the final PCF if burdens from nonused feedstock (e.g., CO_2_ and CO) were allocated to ethylene instead of being burden-free—leading to additional burdens from CO and CO_2_ of at least 0.79 and 1.11 per C_2_H_4_ produced, respectively, as indicated by the LP’s carbon accounting from Table 2 and illustrated in Figure 1.

Besides, byproducts from dehydration, notably CO^28^ (0.19*wt* CO:C_3_H_6_O_2_ and 0.61wt CO:CH_2_O_2_, as showed in Table 2), may pose a challenge to achieving net-zero emissions by partially offsetting (47–51%w*t* C_2_H_4_ and 18–56%*wt* CO generated per CO_2_ used, as accounted in Table 2) sequestration benefits. The mass flow estimates for byproducts were limited to 4 out of more than 40 potential side reactions,^28^ as indicated in eqs S6–S10, potentially underestimating the benefits of CCU. The formation of CO in the PBR can be significant for the PCF. Thermal kinetics indicated that ethyl formate would generate 0.80*wt* CO: C_2_H_4_ at 425 °C and 0.69*wt* CO: C_2_H_4_ at 375 °C (39 bar).^67^ Process design could lead to lower PCF since uncertainties of the model forecasted average emissions of 0.98*wt* CO: C_2_H_4_ at 250 °C with zeolites (0.38 and 1.59 wt% CO: C_2_H_4_ for LP and HP, respectively). CO, recommended as a precursor of ethylene,^68–71^ could either be sold as a commodity or recycled to enhance factory productivity. In both cases, the advantages might go beyond mitigating the PCF, since the increase in revenues (due to productivity improvements and trading of a new product, CO) and/or green credits (due to avoided emissions resulting from the synergy of non-CO discharge and improvement in productivity) might lead to economic benefits.

Solvent replenishment (ethylene glycol and MeTHF) accounted for ∼20%*wt* of their demand. Ethylene glycol contributed 6% and 19% to PCF_nonoffset_ in HP and LP scenarios, respectively. Only the LP scenario required L–L extraction prior to distillation of the formic acid solution, and its utilization contributed to less than 1% of the PCF_nonoffset_. High-recovery L–L extraction systems could further mitigate emissions by reducing the utility demand in the distillation column. Similarly, auxiliary materials, including glycerol, NaOH, and Na_2_CO_3_ contributed, in total, 2% and 16% to the PCF in the HP and LP scenarios, respectively. Glycerol’s footprint could be reduced by using renewable feedstocks or alternative technologies for its recovery/purification, resulting in up to 30% variability.^72^ Substitution of sodium-based salts with potassium-based salts offers additional opportunities to be explored.^15^

Emissions breakdown indicated that up to 24% and 3% of PCF_nonoffset_ were derived from utilities and electricity, respectively. Higher productivity increased electricity and utilities demand. Electricity use was largely associated with CO_2_ capture, while utility demand depended on process selection. Renewable energy supply justified the low electricity contribution on the PCF for both scenarios.

Complete LCA results (Figure 2b) indicated that ethylene achieved performance similar to that of the benchmark in climate change (PCF) and ozone depletion (ODP) categories. However, other impact categories—acidification (ACP), freshwater eutrophication (FEP), marine eutrophication (MEP), terrestrial eutrophication (TEP), and water use (WUP)—showed substantial increases. This was anticipated given the high consumption of auxiliary materials. Water demand exceeded 4.0 kg/kg MP even under ideal HP conditions. Without wastewater treatment offsets, CCU and benchmark technologies displayed similar performances, reinforcing that low waste generation benefits environmental outcomes.

In short, LCA suggests that the novel ethylene manufacturing process achieves a performance comparable to that of traditional benchmarks regarding GWP and ODP. The reduced carbon footprint of ethylene implies potential revenue opportunities through emissions trading schemes (ETS). Achieving carbon-neutral ethylene demands effective waste treatment, the use of biobased feedstocks, and renewable energy. Nevertheless, reductions in PCF may coincide with increased burdens in other impact categories. LCA revealed significant increases in ACP, ETP, and WUP (primarily due to the use of auxiliary materials). The interplay between the availability of biobased resources, renewable energy, and additional burdens on other impact categories will be pivotal to deploying CCU technologies. Furthermore, improvements in primary data, particularly for low-TRL processes, are vital to minimize uncertainties in predictions.

### 3.2 Techno-Economic Analysis

The average production cost for ethylene was 4.79 ±1.19 USD/kg of MP. The production cost was reduced by improving the operational conditions from the LP to the HP scenario, leading to unit costs of 5.63 and 3.95 USD/kg of MP, respectively. The range of variability of the unit costs indicated that the production of ethylene through the combined CCU and esterification pathway has the potential to compete with ethylene production through the methanol (MTO) or direct routes. The production cost of ethylene directly from flue gas or with methanol as an intermediate was estimated at £3.05/kg in 2019, which would be ∼$3.94/kg in 2024 (estimating an inflation rate of 1.4% from 2019 to 2024 and based on data from Nyhus et al. (2024)). The production costs for ethylene via CO_2_ electroreduction could vary from $3.70 to $4.08/kg of MP.^12^ However, the benchmark market value of ethylene (95% *wt*) was forecast to be 10 -times lower than for production cost via CCU ($0.74–1.01/kg).^73^ In this context, the target production cost might drop to at least 21% of the current analysis to meet the highest market value of $1.01/kg of ref 73. Despite higher yields in the HP scenario, it also resulted in higher CapEx and OpEx than the LP scenario, with NPV< 0. NPV, CapEx, and OpEx for both LP and HP scenarios are depicted in Figure 3.

**Figure 3:**
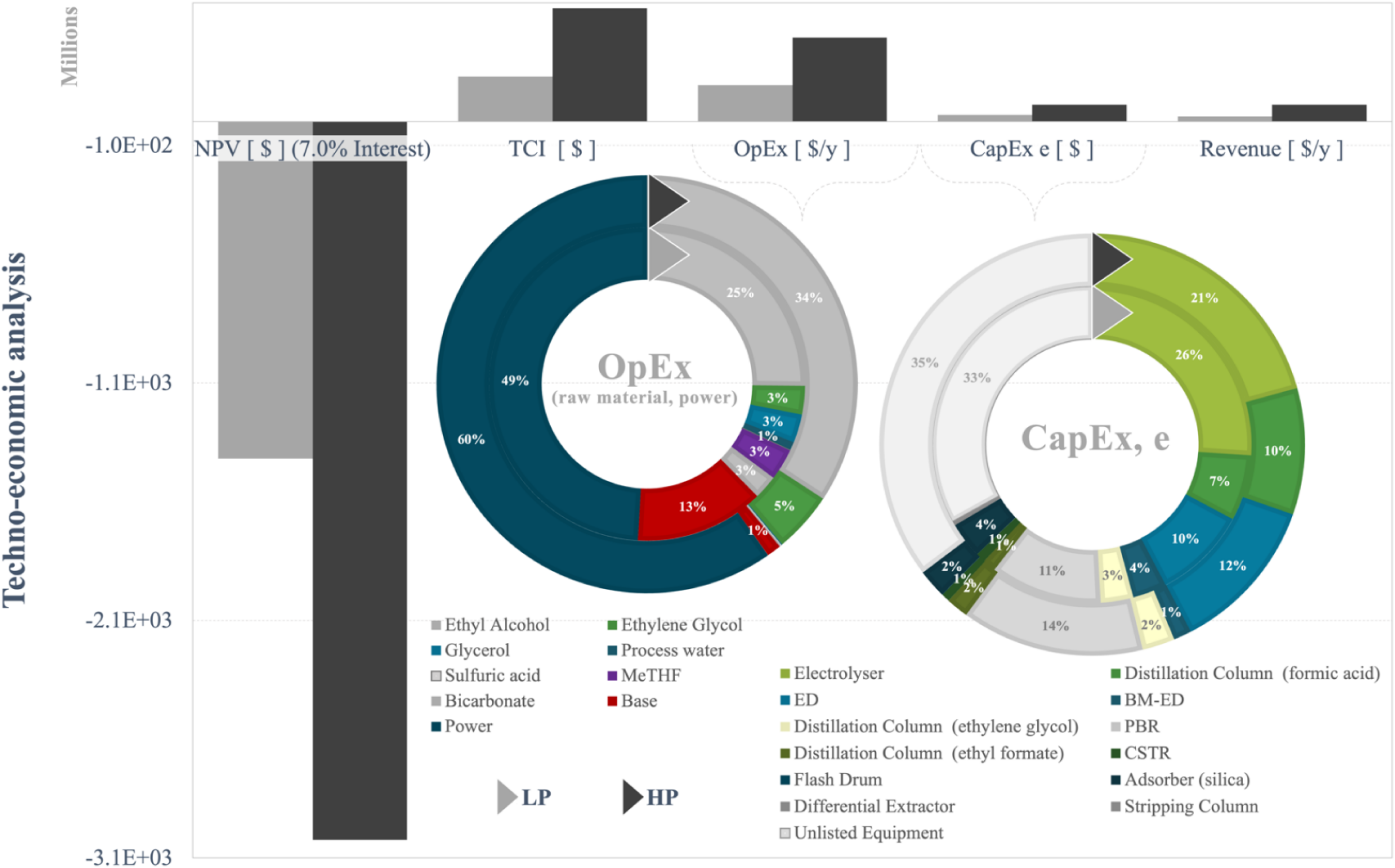
TEA and costs breakdown per MP. BFG flow rate did not add assets to the factory.

The demand for power and raw materials, estimated at least 33% and 26%, respectively, constituted the most significant costs. Together, these costs were up to 70% of the total OpEx. Increasing productivity in the electrolyzer increased the power demand by 11% in the ethylene factory, as indicated in Figure 2. The variation in power demand can be explained by two factors. First, the higher the productivity in the electrolyzer, the higher the electricity demand to reduce CO_2_ into formic acid. Alongside this, the combined L–L extraction and distillation process to recover formic acid in the LP scenario was more efficient than the single distillation arrangement. The OpEx breakdown showed that the HP scenario had 6% more OpEx from utilities than the LP.

Operating under high-efficiency conditions in the HP scenario (low losses, high recycle rates, and low utilization of water) reduced auxiliary material costs from 26% to 6%. However, the impact on OpEx due to energy consumption surpassed the economic benefit of reducing the raw material costs by 20% in the HP scenario. A significant increase in the demand for ethyl alcohol (9%) was observed with improvements in Carbon Capture Utilization (CCU) yield, an expected trend in the M&EB after increasing the formic acid flow rate.

The variation in equipment capital expenditure (CapEx^E^) represented approximately 15% of the total capital investment (TCI). The electrolytic system and distillation columns (ethyl formate, solvent recovery, and formic acid) were the key contributors to CapEx^E^, constituting at least ∼10% of CapEx^E^.

Indeed, the deployment of CCU technologies might require extra revenues. ETS, charging for the treatment of industrial flue gases, or considering the biorefinery concept to integrate CCU technologies into existing industrial sites are alternatives to achieve both net-zero emissions in the ethylene supply chain and profitable businesses.

### 3.3 Sensitivity and Uncertainty Analyses

The global sensitivity analysis forecasted the impact of uncertainties in ethylene production by applying a combined TEA-LCA approach. Uncertainties were limited to the M&EB of LP (lower boundary) and HP (upper boundary) inventories.

The environmental analysis focused on the M&EB, accounting for avoided emissions from CO_2_ captured within CCU process. For operational conditions that provided emissions lower than the benchmark, the economic benefits of green certification under ETS were considered. Emissions were compared against the benchmark to assess the probability of carbon offsetting via PCF_Net_. Global sensitivity analysis and uncertainty for the PCF were summarized in Figure 4a ,b, respectively.

**Figure 4:**
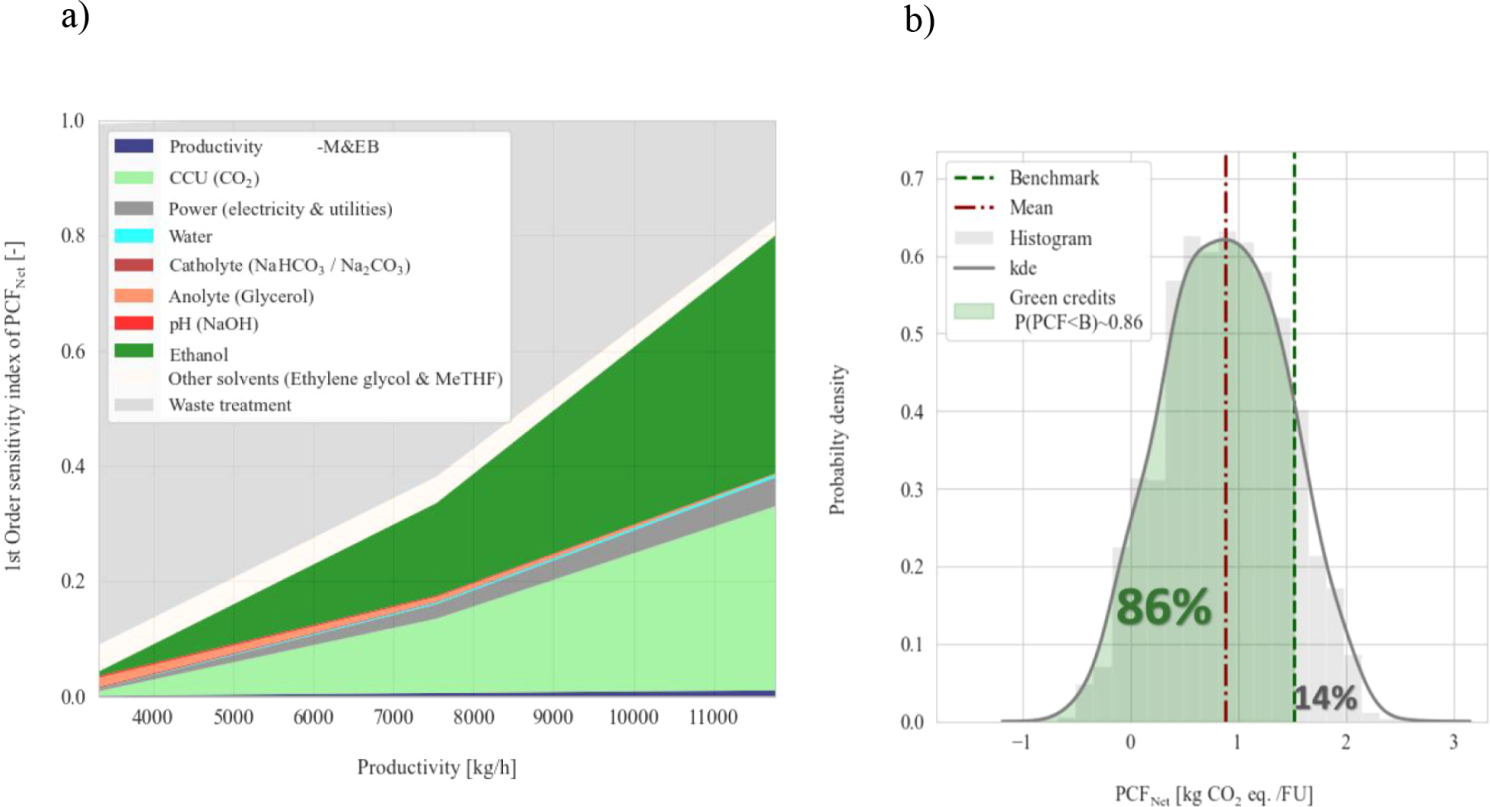
Global sensitivity and uncertainty analyses for PCF. First-order Sobol index (impact proportional to graph’s area) (a). Probability density, with the probability of green certification shaded in green (b).

Uncertainty in the CCU capacity introduced greater variability in the PCF than productivity fluctuations. Productivity uncertainty (±10%) resulted in minimal impact on the PCF (∼1%). Sensitivity analysis revealed that the PCF contribution increased proportionally to productivity (Figure 4a). A 30% variation in CCU capacity led to approximately, 1–31% variability in PCF, which was also amplified under high productivity conditions. Auxiliary materials and power were the primary sources of uncertainty.

Auxiliary material consumption varied significantly between scenarios: ethanol (0.7–40%), bicarbonate (0.03–0.2%), glycerol (0.1–1.6%), and solvents (ethylene glycol, MeTHF, 2.6–4.6%). Except for wastewater treatment, auxiliary materials usage followed an analogous trend: the higher the productivity, the higher the impact on the PCF. This result suggested that material use had a greater impact on PCF than product yield or avoided emissions. In the HP scenario, credits from wastewater treatment were lower than those in the LP scenario due to extensive recycling rates.

Solvent usage (e.g., glycerol, ethylene glycol, and MeTHF) in purification processes decreased due to mass-energy transfer and thermolytic aspects in solvent recovery systems. Enhancing solvent recovery or replacing them with greener alternatives could reduce the PCF of ethylene by up to 30% in the LP scenario. MeTHF, for instance, was identified as a greener solvent alternative to THF because its production uses renewable sources [25]. THF was used to estimate emissions of MeTHF in the background process due to lack of data for MeTHF, while costs, efficiency, and operational conditions of the extraction and stripping column were estimated for utilization of MeTHF.^49^ Solvent replenishment was ∼20% of the total demand.

Ethanol consumption during the esterification process was a major contributor to the PCF uncertainty. As productivity increased, so did the contribution of ethanol, as shown in the shaded area of Figure 4a. This trend was expected since the increase in the productivity of ethylene required an additional ∼3-fold annual amount of ethanol.

Power demand had less impact on PCF than material use since only a renewable energy source was considered. Transitioning to renewable energy grids is crucial not only for the esterification pathway but also for CCU competitiveness overall. For instance, in the cascade conversion of CO_2_ into CO in a direct route, the PCF of producing ethylene in alkaline flow cell technologies can vary considerably by exchanging power sources (with PCF ranging from −3.1 to 58.0 kgCO_2**eq /**_kg FU.^74^ The large variation in PCF indicates the importance of careful selection of operational conditions in forecasting carbon offsetting probabilities by using sensitivity analysis. This is key for stakeholders in making investment decisions related to the proposed esterification pathway to produce ethylene, since under certain operational conditions within the study, carbon offsetting may not be possible.

In fact, achieving net-zero targets with CCU strategies remains challenging when multiple downstream processes are required to recover ethylene. The uncertainty analysis (Figure 4b) indicated ∼86% probability of achieving lower emissions than the benchmark. Although the PCF of the benchmark was fixed, this can also vary due to different feedstocks, technologies, and/or renewable auxiliary inputs utilization. For instance, the use of renewable energy (wind) instead of coal in ethylene production with the benchmark technology (Jung et al., 2024) reduced the PCF of ethylene by 80%. Here, up to ∼78% of ethylene’s benchmark footprint was mitigated, indicating a risk of not offsetting emissions against another benchmark. Thus, securing green credits under ETS schemes would require consistent outperformance against a specific technology for benchmarking emissions.

Global sensitivity analysis and uncertainty of NPV (Figure 5a b) incorporated ETS assets. OpEx contributed over 90% of the NPV variability (Figure 5a), making it the dominant source of uncertainty. Revenue, TCI, productivity, and green credits price were less significant, accounting up to 10%.

**Figure 5:**
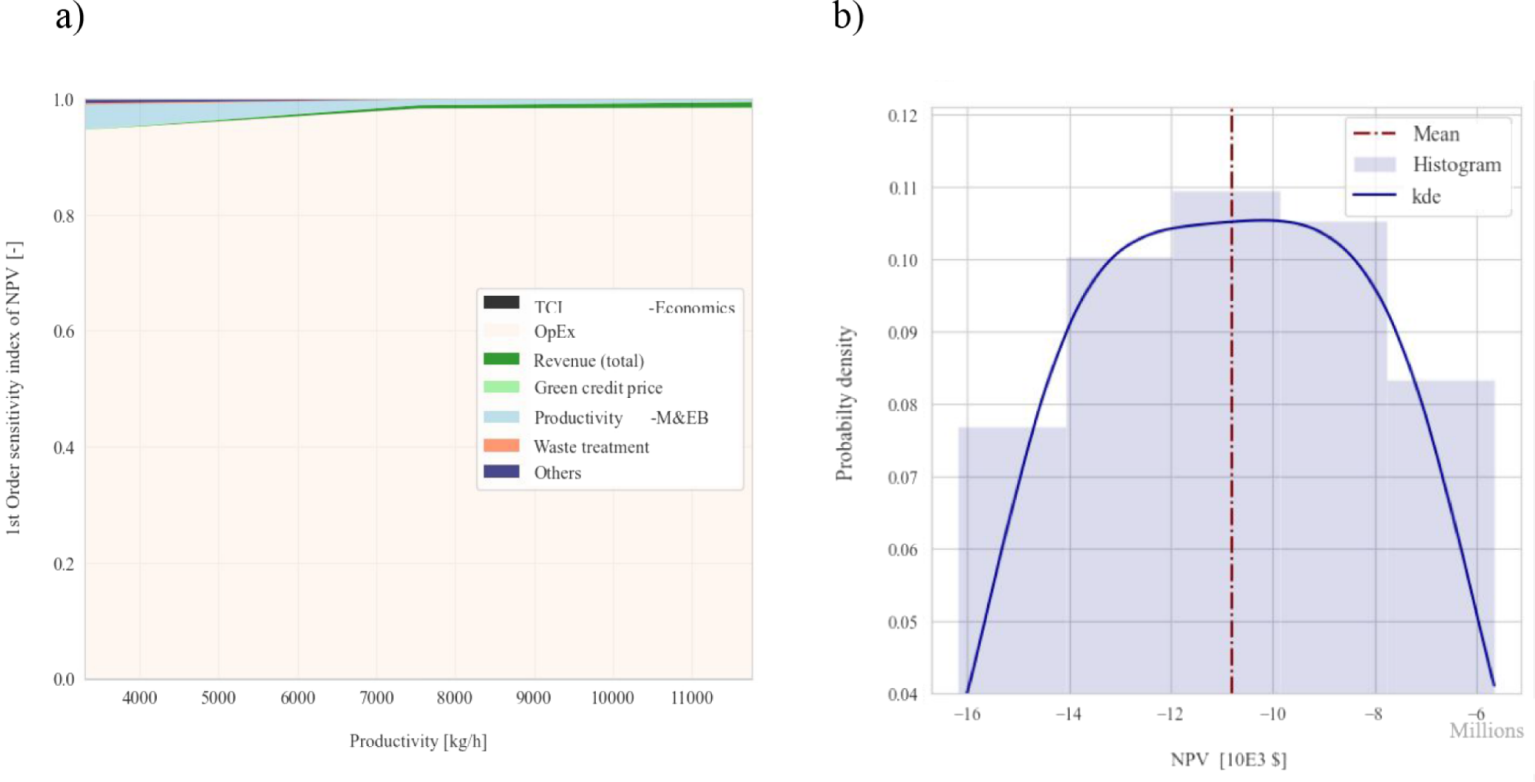
Global sensitivity and uncertainty analyses for NPV—ETS assets included. First-order Sobol index (a). Probability density (b).

The OpEx breakdown (Figure 6a) revealed that energy demand (power + utilities) and raw materials use contributed 35% and 44%, respectively. Energy recovery through heat exchangers could reduce steam demand, which varied from 49 to 65% (Figure 6b). Reducing raw materials cost might be challenging (Figure 6c) since ethanol (up to 35%) is indispensable for the esterification reaction. The pH control at the electrolytic cell explained up to 13% (LP scenario) of OpEx and could be reduced by mitigating losses and operating with less diluted systems (HP scenario in Figure 6c). The recovery of solvents in the process might also be optimized to reduce their significance on OpEx (∼5%). Solvent replenishment should target less than 20% of the total demand to move toward a profitable CCU factory.

**Figure 6:**
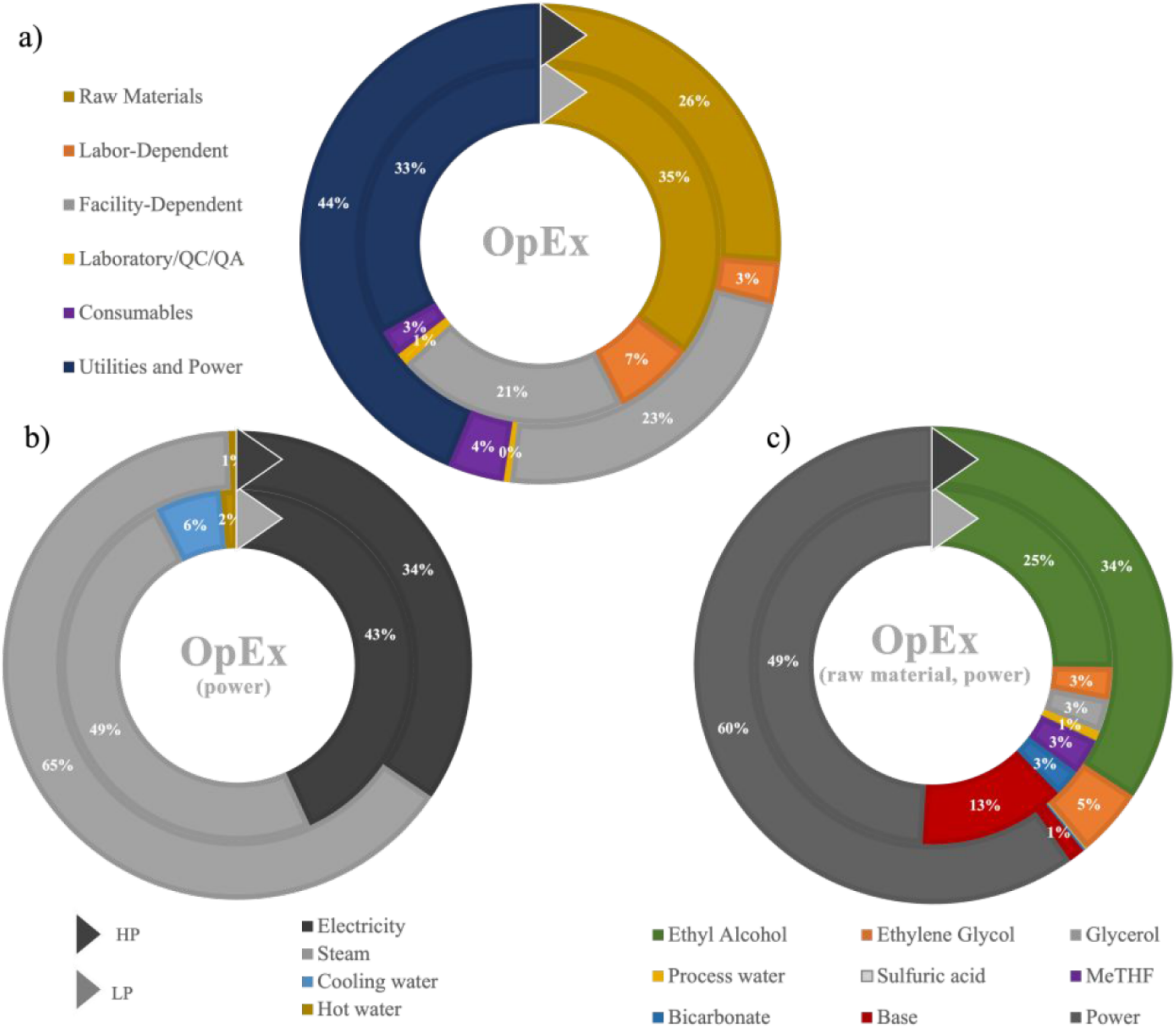
OpEx breakdown across uncertainty ranges for the LP and HP conditions. Total (a), power (b), and raw materials (c).

Ultimately, the deployment of CCU technologies might require not only ETS but also the creation of a new supply chain for treating industrial flue gases with low-grade CO_2_ content, which would increase assets and potentially lead to profitable scenarios.

## 4 Conclusions and Recommendations

Directions for future optimization of ethylene production were guided by a combined TEA-LCA and SA-UA framework, comparing the novel production route (via CO_2_ electroreduction, esterification, and dehydration reactions) with an established benchmark. High productivity during the CO_2_ electroreduction increased both CapEx and OpEx. In relation to CapEx, developing efficient purification steps for diluted streams might be crucial for achieving ethylene decarbonization. OpEx was the largest contributor to NPV variability, emphasizing the importance of managing power and raw material costs for profitability. Global sensitivity and uncertainty analyses suggested potential for low-to-zero PCF in ethylene production. Future research should focus on minimizing design complexity by reducing byproduct formation, controlling the replenishment of auxiliary materials, and prioritizing renewable power and biobased auxiliary materials. The supply chain design will be critical since, in a circular economy context, the utilization of renewable resources could be problematic due to availability, cost, and additional environmental burdens. The key recommendation is to conduct comprehensive LCA rather than focusing exclusively on PCF. Moreover, uncertainties could be minimized by using primary data from semipilot or larger-scale production to enhance data quality and reliability.

## Supplementary Material



## Data Availability

All data that support the findings of this study were provided.

## Notes

Abbreviations; inventory and modeling assumptions for the Ethylene factory (reactions, operational conditions and equipment definition, and process diagram); techno-economic estimates (assumptions, default parameters); background processes (reference for the inventory from Ecoinvent database); deviation range for UA–SA (variability ranges for uncertainty and sensitivity analysis); results; references; graphical abstract; list of supplementary tables ; and list of supplementary figures. (PDF)
