# Supplementary material for: Designing the Ethylene Factory for Products of Carbon Dioxide Reduction: Techno-Economic and Life Cycle Assessments
Source: ACS Sustain Chem Eng. 2025 Sep 11;13(37):15257–68. doi: 10.1021/acssuschemeng.4c10485 (PMC12458976; doi:10.1021/acssuschemeng.4c10485)
Supplement: Supplementary file 1 [file sc4c10485_si_001.pdf]

SUPPORTING INFORMATION

DESIGNING THE ETHYLENE FACTORY FOR PRODUCTS OF CARBON DIOXIDE REDUCTION: TECHNO-ECONOMIC AND LIFE CYCLE ASSESSMENTS

Ariane Silveira Sbrice Pinto[1 +*],

Nalan Gulpinar[1], Fang Liu[1], Elizabeth A. Gibson[2], Linsey Fuller[3], Philip Souter[3].

[1] Business School, Management Department, Durham University, DH1 3LB, Durham, England, United Kingdom.

[2] Energy Materials Laboratory, Chemistry, School of Natural and Environmental Science, Newcastle University, NE1 7RU, Newcastle-upon-Tyne, England, United Kingdom.

[3] Procter and Gamble, Newcastle Innovation Centre, Whitley Road, Longbenton, Newcastle upon Tyne NE12 9TS, United Kingdom.

+ First author.

*Corresponding author.

E-mail: sbrice.a@pg.com; assp_sbrice@outlook.com.

Content

ABBREVIATIONS 3

1. **Inventory and modelling assumptions** 4

    1.1. **The Ethylene factory** 4

    1.2. Techno-economic estimates 14

    1.3. Background processes 17

    1.4. **Deviation range for UA-SA** 18

2. **Result's data** 19

3. **Graphical abstract** 22

REFERENCES 23

LIST OF SUPLEMENTARY TABLES 30

LIST OF SUPLEMENTARY FIGURES 30

ABBREVIATIONS

ACP	,	Acidification [mol H^+ $_{eq}$]
ER, n-r	,	Energy resources, non-renewable [MJ]
FEP	,	Freshwater eutrophication [kg P $_{eq.}$]
FWP	,	Freshwater ecotoxicity [CTUe]
FWPi	,	Freshwater ecotoxicity, inorganics [CTUe]
FWPm	,	Freshwater ecotoxicity, metals [CTUe.]
FWPo	,	Freshwater ecotoxicity, organics [CTUe]
GWPb	,	Climate change, biogenic [kg CO_2 $_{eq.}$]
GWPf	,	Climate change, fossil [kg CO_2 eq.]
GWPlluc	,	Climate change, LLUC [kg CO_2 eq.]
HTPc	,	Human toxicity, cancer [CTUh]
HTPc,m	,	Human toxicity, cancer, metals [CTUh]
HTPc,o	,	Human toxicity, cancer, organics [CTUh]
HTPnc	,	Human toxicity, non-cancer [CTUh]
HTPnc,m	,	Human toxicity, non-cancer, metals [CTUh]
HTPnc,o	,	Human toxicity, non-cancer, organics [CTUh]
IRP	,	Ionising radiation [kBq U235]
LUP	,	Land use [pt]
MEP	,	Marine eutrophication [kg N eq.]
ODP	,	Ozone depletion [kg CFC-11 eq.]
PCF	,	Climate change (GWP 100a) [kg CO_2 $_{eq.}$]
PMP	,	Particulate matter [Disease incidences]
REPm	,	Material resources, metals/minerals [kg Sb eq.]
TEP	,	Terrestrial eutrophication [mol N eq.]
WUP	,	Water use [m^3 water eq. of deprived water]

1. Inventory and modelling assumptions

1.1. The Ethylene factory

Ethylene can be produced from CO_2 via intermediate chemicals such as formic acid and esters (e.g., ethyl formate). In CCU factories, CO_2 is an important feedstock that could be recovered from industrial flue gases. The blast furnace gas (BFG) is an example of flue gas generated in steel production, which contains up to ~36% of CO_2 (*wt*) in its composition. A typical steel factory produces 9,000 tonnes of steel/day[1], which generates ~2.5 tonne of BFG/tonne of steel. CCU sites attached to this industrial site could have high feedstock availability, ~900 tonne of BFG/h. Other flue gases sources also include the burning of coal, crude oil, and natural gas. Table S1 compares the composition of potential flue gases to be used as feedstocks for chemicals production in CCU factories.

Table S1- Mole fraction of fuels.

Fuel	Mole fraction (c_i) [mol%]							References
	C	H	N	O	S			
Coal	51.2	43.0	0.9	4.2	0.7			[2]
Oil	38.2	61.3	-	-	0.5			[3]
**Emissions**	**CO**	**H_2**	**N_2**	**CO_2**	**CH_4**	**C_2H_6**	**H_2O**	
NG	-	-	-	-	100	-	-	
BFG	23.9	4.4	49.0	22.7	-	-	-	[4]
BOFG	65.2	0.7	18.5	15.6	-	-	-	[4]
COG	6.7	55.6	5.7	2.4	26.9	1.2	-	[4]
Syngas (Lignocellulosic materials)		30-60	25-30	-	5-15	0-5	-	[5]

NG: natural gas, BFG: blast furnace gas, BOFG: basic oxygen furnace gas, and COG: coke oven gas.

The model ethylene factory used ~100 ton/h of BFG to produce ethylene (98% *wt*). Two scenarios were used to evaluate uncertainties in modelling TEA and LCA. The first scenario (LP, or scenario 1) considered low productivity. The operational conditions of LP were based on the current technology readiness level (TRL), rated as 4. The second scenario considered high productivity (HP, or scenario 2). For HP, potential improvements with the technology deployment over time were evaluated. Figure S1-a and -b display the process diagram for each scenario.

Formate/formic acid from CO_2 was produced in an electrocatalytic cell (P-1) in the CCU system and recovered as the acid in the formic acid system. The source of NaOH was the anolyte chamber (P-2), while CO_2 from the industrial flue gas was absorbed with carbonate solutions in the catholyte (P-3) [6].

The overall stoichiometry of carbonates absorption was shown in Eq. (S1) [7].

$$Na_2CO_3 + H_2O + CO_2 \rightleftharpoons 2NaHCO_3 \quad \text{Eq.(S1).}$$

Experimental data have shown variations from 10-40%*wt* in carbonates to bicarbonates conversion [8] due to mass transfer limitations. The maximum conversion of Na_2CO_3 (10% *wt*) at 30 °C was 70% [7]. The electrolyser of LP operated at 70 °C with a concentration below 0.1% *wt* CO_3^{-2} - HCO_3^-, where low conversion of carbonate to bicarbonate might occur [8]. Here, the CCU factory operated with 20% and 40% of Na_2CO_3 to $NaHCO_3$ conversion for LP and HP scenarios, respectively. This estimate might be sensible since, depending on the catalyst, high $KHCO_3$ concentration can favour formate generation. Conversely, the application of acidic or basic binders had a negative impact on formate yields[9].The mass-transfer impact was neglected.

Although LP and HP boundaries considered potassium carbonate kinetics [8], variations in Henry's constant of potassium and sodium carbonate were not meaningful [10] for aqueous solutions with low carbonate concentration (<1 M, T=50 °C) [10]. The solubility of CO_2 into 5%*wt* carbonate solutions at 25 °C varied from 0.01575 (T=50 °C) to 0.02412 (T=25 °C) mol L^{-1} bar^{-1} [8], which could also be used to limit the concentration of CO_2 in the catholyte. Although the presence of H_2/CO in the BFG could gradually decrease the electrolyser productivity over time in formate electrolysers (e.g., up to 20% in 20h for Pd on activated carbon[11]), these inefficiencies were neglected in all scenarios of our study.

In the electrolytic cell (P-1/EC), the overall equation of formic acid production is shown in Eq. (S2).

$$CO_2 + 2H^+ + 2e^- \rightarrow CO_2H_2 \qquad [E^o = -0.20 \text{ V vs SHE}] \qquad \text{Eq.(S2)}$$

The productivity of formate/ formic acid in the electrolyser reached ~0.024M/h (1.2 M in 50h [6]) for the LP scenario, which led to ~30% of the theoretical yield ($CO_2H_2/CO_{2\,(g)} \cong 1.046\ w{:}w$). The yield for the HP scenario was estimated as ~75% of the theoretical. The power demand of this system varies in the range of 1.36-7.92 kW/g of formic acid/h [6],

depending on inefficiencies in the system (e.g., ohmic resistance, side reactions to produce hydrogen, oxygen and other products).

The oxygen evolution reaction (OER) was identified as a common reaction in the anodic compartment (P-02) across various reactor configurations for the continuous electroreduction of CO_2 to $HCOO^-$ and HCOOH [12]. OER is generally perceived as a sequence of single electron/proton charge transfer reactions [13], and the overall reaction was shown in Eq. (S3).

$$H_2O \rightarrow 1/2O_2 + 2\,H^+ + 2e^- \qquad [E^O = 1.23V \text{ vs SHE}] \qquad \text{Eq.(S3)}$$

To mitigate the hydrogen evolution reaction (HER), the electrochemical reduction of CO_2 in aqueous media is commonly conducted under alkaline conditions. Neutral pH of the electrolyser was controlled with NaOH. Formic acid dissociated into formate at pH ~3.45, then, the production of the organic acid was neglected. The anolyte (P-02) solution was an aqueous mixture of NaOH [3M] and glycerol (polyol) [6]. Auxiliary chemicals [6] such as polyol stabilizers (hydrides, halides, phosphines, porphyrins) and/or metals (catalysts as Pb) were not included in the calculations. The CCU technology was rated at 4 in the Technology Readiness Level (TRL) scale [14]. Further details about the technology were published by Pinto *et. al*[15].

Technologies downstream of CCU technologies [16] included degasification (P-13), membrane separation (P-5)/concentration (P-8) of formic acid, L-L extraction (P-7, only for LP scenario), and distillation (P-11). The de-gasification was important to purge remaining gases of the liquid flow. To recover an aqueous solution of formic acid from formate, NaOH was removed/recycled using bipolar membrane (BPM) electrodialysis (P-05 [17]). Three-fold pre-concentration was considered in a second electrodialysis process (P-08 [18]). Since the distillation column required a feed stream with at least ~30% of formic acid, liquid-liquid extraction (P-07) with 2-methyltetrahydrofuran (MeTHF) was considered [19] for the LP

scenario, since the concentration of formic acid was below the ~30% target. MeTHF was recovered in stripping columns (P-12) and recycled. The replenishment of MeTHF was, approximately, 20% of the total demand. The formic acid (methanoic acid) was purified up to 65%*wt* grade by distillation (P-11) to be used as the precursor in ethylene production through the esterification pathway[20].

The esterification reaction [21,22] occurred by reaction of ethyl alcohol and formic acid forming an ethyl formate. The catalyst was sulfuric acid (1% of amount of formic acid) [23], as shown in Eq. (S4).

$$CH_2O_2 + C_2H_6O \xleftrightarrow[H_2SO_4]{} C_3H_6O_2 + H_2O \qquad (T=70\ °C) \qquad \text{Eq. (S4)}$$

The process for manufacturing ethylene from ethyl esters was modelled by a simplified system containing a continuous stirred tank (CSTR, P-25), and an extractive distillation system (P-21; P-28). In the continuous stirred tank (CSTR, P-25), ethanol (92% *wt*) reacted with formic acid to produce ethyl formate. [23]

Due to the formation of an azeotrope mixture, the extractive distillation (P-21) was used to separate ethyl formate. Effective extraction agents for water, alcohols and/or esters mixtures are ethylene glycol, propylene glycol, butanediol and/or glycerol [24]. Ethylene glycol was used to design the extractive distillation [25]. Ethylene glycol was recycled by distillation (P-28). The replenishment of the solvent was up to ~16% of the total demand (P-30).

Manufacturing ethylene from the ethyl ester [20,26] was done in a packed bed reactor (PBR, P-35) at 250 °C with zeolite as catalyst [20]. Although the kinetics of reactions on zeolites were not described by Cognion-Durual (1984) [20], the degradation kinetics of ethyl formate in high temperatures was reported by Balla et al. (2017) [27]. The thermal decomposition of ethyl formate leads to the formation of ethylene mixed with a significant quantity of by-products.

Formic acid, ethanol, ethane, carbon dioxide, carbon monoxide, and water were byproducts of the degradation/cracking of ethyl esters [20,26]. The unimolecular elimination of ethylene from ethyl formate is the primary decomposition reaction, as indicated in Eq. (S5) [27,28].

$$C_3H_6O_2 \underset{\Delta}{\longrightarrow} C_2H_4 + CH_2O_2 \quad k_1 = 2.18\text{x}10^{12}\exp\left(-\frac{48.1}{RT}\right) \qquad \text{Eq. (S5)}$$

The conversion of 100% of ethyl formate was obtained with selectivity in ethylene of 98% when the cracking was catalysed by zeolites [20]. Among 43 potential by-products [27], the final product contained a mixture of ethylene, carbon monoxide, and ethane. The stoichiometry of Eq. (S6) to (S10) included the production of ethanol, ethane, carbon dioxide, carbon monoxide, and water as the key by-products[27].

Ethanol generation:

$$C_3H_6O_2 \rightarrow C_2H_6O + CO \qquad \text{Eq. (S6)}$$

Ethylene production from ethanol:

$$C_2H_6O \rightarrow C_2H_4 + H_2O \qquad \text{Eq. (S7)}$$

CO production:

$$CH_2O_2 \rightarrow CO + H_2O \qquad \text{Eq. (S8)}$$

CO_2 production:

$$CH_2O_2 \rightarrow CO_2 + H_2 \qquad \text{Eq. (S9)}$$

Ethane production:

$$C_2H_4 + H_2 \rightarrow C_2H_6 \qquad \text{Eq. (S10)}$$

Considering the complexity of the system, the stoichiometry was simplified by Eq. (S11), which combined Eq. (S5)-(S7) with 98% of selectivity[29] towards ethylene.

$$C_3H_6O_2 \rightarrow C_2H_4 + 1/2\,CO + 1/2\,CCH_2O_2 + 1/2\,CH_2O \qquad \text{Eq. (S11)}$$

Eq. (S8) was used to estimate the decomposition of formic acid. The formation of CO_2 (Eq. (S9)) and ethane (Eq. (S10)) as by-products were neglected, then the final product was a mixture of ethylene and CO, as previously reported as the main by-products [29,30].

The theoretical yield from the primary reaction is 37% wt (Eq. (S5)). For the ethylene factory, the maximum yield of the main product was set at ~90% of the theoretical yield to consider the byproducts formation [27.] The activation energy of producing ethylene can be estimated by 40,010 cal.[30] The formation of CO in the decomposition of ethyl formate can be significant due to impurities or thermal degradation. Under high temperatures, ethyl formate shall generate 0.80 CO : C_2H_4 (*wt*) at 425°C and 0.69 CO: C_2H_4 (*wt*) at 375°C (39 bar). [30] Here, the forecast of ethylene's factory led to 0.38 (LP) up to 1.59 (HP) CO: C_2H_4 (*wt*) at 250°C - including losses. Despite high uncertainty, the thresholds of CO emissions are expected to be lower than the reported. Besides, further experimental data to evaluate the effect of by-pass of the CO in the electrolysis shall lead to lower energy demand and higher productivity in the electrolysis. Although the direct electroreduction of CO_2-CO into C_{2+} [31] products indicated that feeding CO directly in the electrolysis shall benefit the overall performance of CCU, due to poor data availability, this impact was not evaluated in detail for formate's production. Then, to obtain the final product with commercial grade from the mixed by-products flow (S-142 and S-124, for LP and HP respectively), the purification was the next step of the manufacturing process.

Fist, the humidity was removed with adsorption in silica bed (P-38). Next, the gas was purified by following P-34 and P-37. The purification of ethylene has high TRL (~9) since it has been applied in petrochemicals, such as by Chevron Phillips Chemical Corp., LLC. The removal of carbon monoxide from the ethylene flow required high pressure (P-34, 25 bar) and

low temperature (-30 °C) operational conditions in a distillation column (P-37) [32]. Limitations in modelling the gas distillation should be mentioned. The operational conditions of the column were assumed to separate the main product from CO. However, the mass balance reported for the technology indicated 0.01% of CO_2 per ethylene in the feed, while in the ethylene factory it was higher than 50%, resulting from the assumption that the side reactions of the cracking process (P-35) produced mainly CO. Studies suggested that carbon dioxide systems can be highly efficient because they offer a very high refrigeration capacity (22,600 kJ/kg at 0 °C), which varies from 5 to 22 times more than other refrigerants [33]. In subcritical cascade systems [34], CO_2 could be used with a compressor suction temperature of -30/-35 °C, liquifying the feed of P-37. The former property could be very interesting for recovering ethylene in CCU factories due to CO_2 availability, especially through the esterification pathway since it requires low temperature to liquify the feed of gas distillation systems. In this context, the cost of the heating exchange agent in P-37 was neglected.

The design of packed columns used for distillation, gas absorption, PBR, and liquid-liquid extraction was limited by specific constrains[35]. In industrial applications, diameters of fractionation columns vary greatly from 6m to 15m, and the length to diameter ratio (L/D) should be less than 30. Here, the maximum diameter was 8 and L/D $\leq$ 20. The tower height was limited to 60 m to avoid wind, load, and foundation concerns[35].

Overall losses in the ethylene factory were assumed to vary from 0.1 to 10%, as displayed in the process diagram from Figure S1 (a-b).

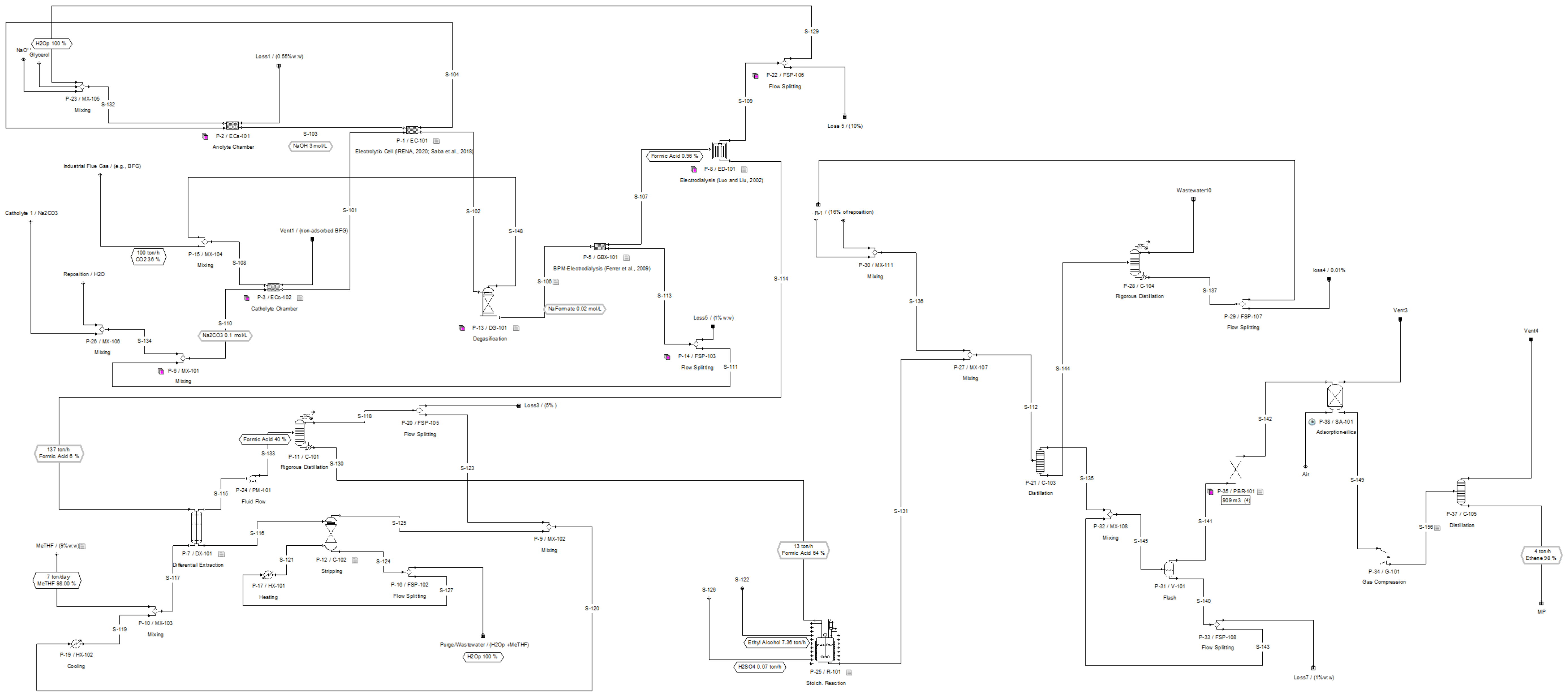
H2Op 100 %
NaOH
Glycerol
P-23 / MX-105
Mixing
S-132
Loss1 / (0.55%w:w)
S-104
P-2 / ECa-101
Anolyte Chamber
S-103
NaOH 3 mol/L
P-1 / EC-101
Electrolytic Cell (IRENA, 2020; Saba et al., 2018)
S-129
P-22 / FSP-106
Flow Splitting
S-109
Loss 5 / (10%)
Formic Acid 0.96 %
P-8 / ED-101
Electrodialysis (Luo and Liu, 2002)
Industrial Flue Gas / (e.g., BFG)
S-101
S-102
S-148
S-107
100 ton/h
CO2 36 %
P-15 / MX-104
Mixing
S-108
Vent1 / (non-adsorbed BFG)
P-5 / GBX-101
BPM-Electrodialysis (Ferrer et al., 2009)
S-106
S-113
S-114
Catholyte 1 / Na2CO3
Reposition / H2O
P-3 / ECc-102
Catholyte Chamber
S-110
Na2CO3 0.1 mol/L
P-26 / MX-106
Mixing
S-134
P-6 / MX-101
Mixing
NaFormate 0.02 mol/L
P-13 / DG-101
Degasification
Loss5 / (1% w:w)
P-14 / FSP-103
Flow Splitting
S-111
Wastewater10
R-1 / (16% of reposition)
P-30 / MX-111
Mixing
S-136
P-28 / C-104
Rigorous Distillation
S-137
P-29 / FSP-107
Flow Splitting
loss4 / 0.01%
Vent3
Vent4
P-27 / MX-107
Mixing
S-144
S-112
Loss3 / (5%)
S-118
P-20 / FSP-105
Flow Splitting
Formic Acid 40 %
S-133
P-11 / C-101
Rigorous Distillation
S-130
S-123
137 ton/h
Formic Acid 6 %
P-24 / PM-101
Fluid Flow
S-115
S-116
P-7 / DX-101
Differential Extraction
S-125
P-9 / MX-102
Mixing
S-121
P-12 / C-102
Stripping
S-124
P-17 / HX-101
Heating
P-16 / FSP-102
Flow Splitting
S-127
MeTHF / (9%w:w)
7 ton/day
MeTHF 98.00 %
S-117
P-10 / MX-103
Mixing
S-119
P-19 / HX-102
Cooling
Purge/Wastewater / (H2Op +MeTHF)
H2Op 100 %
S-120
S-131
13 ton/h
Formic Acid 64 %
S-126
S-122
Ethyl Alcohol 7.36 ton/h
H2SO4 0.07 ton/h
P-25 / R-101
Stoich. Reaction
P-21 / C-103
Distillation
S-135
S-142
P-38 / SA-101
Adsorption-silica
Air
S-149
P-35 / PBR-101
909 m3 (4
S-141
P-32 / MX-108
Mixing
S-145
P-31 / V-101
Flash
S-140
P-33 / FSP-108
Flow Splitting
S-143
Loss7 / (1% w:w)
P-34 / G-101
Gas Compression
S-156
P-37 / C-105
Distillation
4 ton/h
Ethene 98 %
MP

(a)

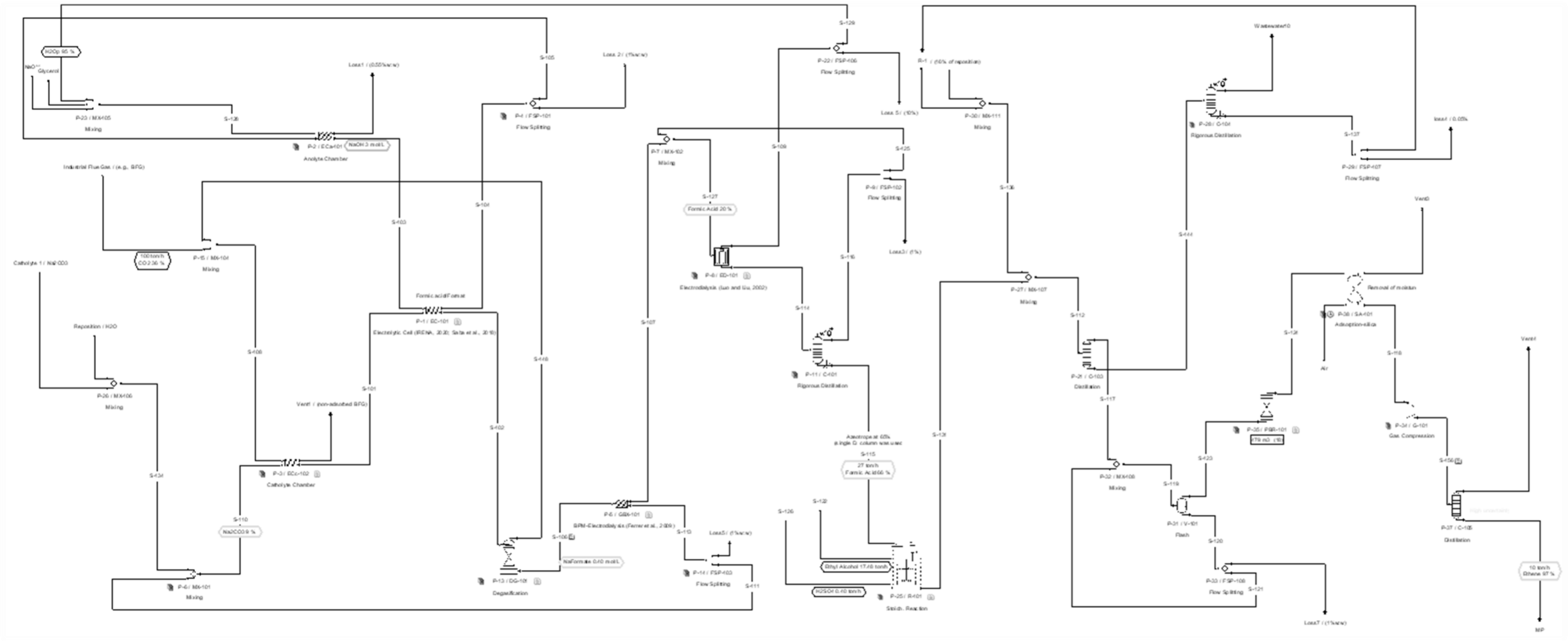

(b)

Figure S1- Process diagram: LP(a) and HP(b).
Convergence errors of 3 to 10% were identified.

1.2. Techno-economic estimates

The complete inventory for both TEA-LCA and SA-UA is summarised in Table S2.

Table S2 - Boundaries for material, energy, and monetary flows used in sensitivity and uncertainty analyses.

Variable	[lower bound, LP,	Mean,	Upper bound, HP]	OpEx/ Revenue	
**Auxiliary Material (inputs) [kg/h]**					
Electrolyte, Na_2CO_3	1.09E+03	6.12E+02	1.36E+02	278.80	USD/ton. (OpEx)
Water, H_2O	3.41E+05	1.96E+05	5.13E+04	0.35	USD/m^3 (OpEx)
Glycerol, $HOCH_2CHOHCH_2OH$	4.54E+02	2.36E+02	1.81E+01	784.66	USD/ton. (OpEx)
pH control, NaOH	3.63E+03	2.18E+03	7.26E+02	444.9	USD/ton. (OpEx)
MeTHF, $C_5H_{10}O$	2.59E+02	1.30E+02	0.00E+00	1560.00	USD/ton. (OpEx)
Catalyst, H_2SO_4	6.35E+01	1.41E+02	2.18E+02	63.51	USD/ton. (OpEx)
Ethyl Alcohol (96%wt), C_2H_5OH	6.68E+03	1.44E+04	2.21E+04	507.00	USD/ton. (OpEx)
Ethylene Glycol, $HOCH_2CH_2OH$	6.65E+03	4.47E+03	2.30E+03	560.00	USD/ton. (OpEx)
**Wastes [kg/h]**					
Aqueous	1.21E+05	7.38E+04	2.71E+04	-	
**Utilities [kg/h]**					
Utilities (total) [a]	1.29E+07	2.94E+07	4.59E+07	0.35	USD/m^3 (OpEx)
Chilled Water	3.57E+06	1.79E+06	0.00E+00		-
Cooling Water	8.89E+06	2.72E+07	4.54E+07		-
Hot Water	3.00E+05	1.50E+05	0.00E+00		-
Steam	1.24E+05	2.96E+05	4.68E+05		-
**Power [kW]**					
Total energy	1.84E+05	3.95E+05	6.07E+05	0.10	USD/ kW-h
Utilities	1.55E+05	3.47E+05	5.39E+05		
Electricity	2.85E+04	4.80E+04	6.75E+04		
**TEA**					
TCI	1.90E+08	3.34E+08	4.78E+08		USD
OpEx	1.54E+08	2.54E+08	3.54E+08		USD/y
CapEx	2.82E+07	4.96E+07	7.11E+07		USD
Revenue (MP)	2.18E+07	4.65E+07	7.12E+07		USD/y

a Only 10%wt of the total was replenished as raw material.

The market prices for utilities were summarized in Table S3.

Table S3 - Physical properties and costs for utilities used in each scenario in SuperPro Process Design ®.

**Utility** [b]	**Physical properties**	**Unit Cost**		**Reference**
**Steam**	242 °C (high P), 152 °C (medium P)	0.0455	USD/ kW-h	[36]
	112 °C (low P)	0.0347	USD/ kW-h	[37]
**Hot Water** [c]	80-43 °C; 1 bar	0.1000	USD/ kW-h	[d]
**Chilled Water**	5-25 °C; 1 bar	0.0295	USD/ kW-h	[38]
	5-10 °C; 1 bar	0.0295	USD/ kW-h	
**Cooling Water**	20-35 °C; 1 bar	0.0172	USD/ kW-h	[39]
	25-60 °C; 1 bar			
**Electricity**	low voltage	0.1000	USD/ kW-h	[40]

[b] Energy recovery was considered for all scenarios.
[c] Based on electricity cost.
[d] SuperPro Process Design ® default.

Table S4- Prices per unit of auxiliary inputs/outputs. Reference year: 2024

**Components**	**Price [$/ Unit]**	**Unit**	**Reference**
**Output/ Product**			
Ethylene (95% *wt*)	795	ton	[41]
**Inputs**			
CO_2, CO, H_2 BFG	-	Nm^3	-
Ethylene glycol	560	ton	-
Glycerol	784.66	ton	[42]
Ethanol (96% *wt*)	507	ton	[43]
Na_2CO_3	278.80	ton	[44]
NaOH	444.90	ton	[45]
MeTHF	1,560	ton	
Water process	0.35	m^3(STP)	13
BFG (based on green credits price)	100	ton	[46–48]

Table S5- Equipment summary.

Name	Type	Units		Required Capacity [e]			Notes [e]		Unit Price (USD/Unit)		
		LP	HP	LP	HP		LP	HP	LP	HP	Mean
EC-101	Electrolytic Cell	- [f]		24.00	48.00	MW	15 ton. Formate/h (~1.00 g/L); T=30 ºC	47 ton. Formate/h (~4.13 g/L)	7.35E+06	1.47E+07	1.10E+07
G-101	Centrifugal Compressor	1	2	2.04	3.52	MW	D P=24 bar		3.24E+06	5.38E+06	4.31E+06
C-101	Distillation Column	1	1	4.38E+02	2.51E+03	m^3	P=2 bar (design); H/D=4.80; D=5m; 30 stages	P=2 bar (design); H/D=6.25; D=8m; 40 stages	1.88E+06	6.82E+06	4.35E+06
ED-101	Electrodialyser	2	4	1.07E+04	3.34E+04	m^2	534 stacks; 10 m^2 per pair	835 stacks; 10 m^2 per pair	1.35E+06	2.12E+06	1.74E+06
BPM-101	Electrodialyser	1	1	1.62E+04		m^2	Adiabatic ; T=32 ºC ; 0.20 MW; 40% of filtrate recovery; 0.4 A/m^2		9.72E+05	9.72E+05	9.72E+05
C-104	Distillation Column	1	1	6.20E+01	2.13E+02	m^3	P=1.5 bar (design); H/D=6.60; D=2.25 m; 38 stages	P=1.5 bar (design); H/D=3.60; D=4.22 m; 38 stages	9.24E+05	1.74E+06	1.33E+06
SA-101	Adsorber (for Gaseous Streams)	2	1	1.30E+02	8.61E+02	m^3	Depth/D =1 ; D=5.49 m	Depth/D =1 ; D=10.31 m	8.06E+05	1.56E+06	1.19E+06
PBR-101	Packed Bed Reactor	4	11	9.09E+02	1.10E+03	m^3	H/D=15 ; H=60 m	H/D=10 ; H=52 m	7.99E+05	8.99E+05	8.49E+05
C-103	Distillation Column	1	1	3.14E+01	49.46	m^3	P=1.5 bar (design); H/D=5.00; D=2.00 m; 33 stages	P=1.5 bar (design); H/D=6; D=2 m; 33 stages	5.97E+05	1.20E+06	8.99E+05
R-101	Stirred Reactor	1	1	2.09E+01	66.96	m^3	H/D=2.50 ; H=5.50 m	H/D=2.50 ; H=8.11 m	2.76E+05	4.65E+05	3.71E+05
HX-102	Heat Exchanger	1	[g]	2.90E+02	-	m^2	T=40 ºC (exit)	-	1.84E+05	-	-
C-105	Distillation Column	1	1	4.51E+00	1.03E+01	m^2	H/D=4.00 ; H=4.00 m	H/D=2.00 ; H=4.00 m	1.22E+05	1.54E+05	1.38E+05
DX-101	Differential Extractor	2	[g]	1.89E+01	-	m^3	H/D=3.00; D=2 m	-	1.21E+05	-	-
HX-101	Heat Exchanger	1	[g]	1.47E+02	-	m^2	T=125 ºC (exit)	-	1.19E+05	-	-
C-102	Stripper	1	[g]	5.12E+01	-	m^3	H/D=2.00; D=3 m	-	5.90E+04	-	-
PM-101	Centrifugal Pump	1	[g]	9.90E-01	-	kW	-	-	2.60E+04	-	-
V-101	Flash Drum	1	1	2.20E-01	7.20E-01	m^3	T=60 ºC		9.00E+03	1.50E+04	1.20E+04

[e] The selection of the equipment and the respective scale was based on the literature review. The key experimental data was used to model the process in SuperPro Process Design ® software. The optimization of the parameters would improve the resolution of the model. The following proportions were set as max.: L/d<30 m; 8 m<D<15 m; and H<60 m- as described in in section 1.1 The ethylene factory in SI.

[f] Only the energy demand was designed to estimate the CapEx of the entire system (306 USD/kW).

[g] The solvent extraction was removed from the HP scenario since the concentration was enough for the distillation input. However, the capacity of the distillation and the energy demand increased.

1.3. Background processes

The list of background processes for life cycle assessment is provided in Table S6.

Table S6- LCA's Inventory per functional unit (FU, 1 kg of ethylene).

Foreground	Amount	Background (Ecoinvent, cutoff)		Unit
		Reference	Region	
**Inputs**				
Carbon dioxide	3.14	-	-	kg/kg FU
Hydrogen (BFG)	0.03	-	-	kg/kg FU
Impurities (gaseous)	10.88	-	-	kg/kg FU
Glycerol	0.00	glycerine production, from epichlorohydrin	RER	kg/kg FU
(Bi)carbonate	0.01	sodium bicarbonate, to generic market for neutralising agent	GLO	kg/kg FU
Alkaline solution	0.06	soda production, Solvay process	RER	kg/kg FU
Water (process)	4.54	market for tap water	Europe without Switzerland	kg/kg FU
Ethyl alcohol	1.96	ethanol production from sugar beet	RoW	kg/kg FU
Utilities (replenishment, 10%wt)	405.91	market for tap water	Europe without Switzerland	kg/kg FU
Ethylene glycol (replenishment)	0.27	ethylene glycol production	RER	kg/kg FU
Methyl tetrahydrofuran (replenishment)	0.00	tetrahydrofuran production	RER Europe	kg/kg FU
**Outputs**				
Ethylene	1.00	ethylene production, average (baseline)	RER	kg/kg FU
Oxygen (OER)	0.70	-	-	kg/kg FU
Ethyl formate (loss)	0.09	-	-	kg/kg FU
**Wastes**				
Solid waste	0.00	treatment of municipal solid waste, incineration	GB	kg/kg FU
Aqueous waste	2.40	treatment of biowaste by anaerobic digestion	RoW	kg/kg FU
**Emissions**				
Carbon monoxide (BFG, non-captured)	0.79(HP)-7.33(LP)		RER	kg/kg FU
Carbon dioxide (BFG, non-captured)	1.11 (HP)-3.87(LP)	Carbon monoxide/dioxide, fossil, non-urban air or from high stacks		
Carbon dioxide (captured)	2.02			kg/kg FU
Carbon monoxide (by-product from dehydration)	0.38 (LP)-1.59 (HP)		RER	kg/kg FU
**Power demand**				
Heat	47.70	electricity production, wind, >3MW turbine, onshore	GB	kWh/kg FU
Electricity	5.97		GB	kWh/kg FU

It is worth noting that because of CO's short lifetime, its GWP can be significantly higher for shorter time horizons (e.g., 20 years) than over a 100-year period. This is because the gas is more effective at trapping heat in the short term, but its impact diminishes faster as it breaks down. Estimated indirect Global Warming Potentials for CO for time horizons of 20, 100, and 500 years indicated the following ranges: 2.8 -10.0; 1.0 - 3.0; and 0.3-1.0, respectively[49]. Table S7 shows different time horizons for CO's GWP forecast.

Table S7- *Indirect GWP for CO in different time horizons* [49].

Assumptions for different models	***GWP for a specific time horizon [kg $CO_{2\ eq.}$ / kg CO]***		
	20 years	***100 years***	***500 years***
Box model including CH_4 feedbacks only	*2.8*	*1.0*	*0.3*
2-dimmentional model with CH_4 feedbacks and tropospheric O_3 production by CO	*10*	*2.2 to 3.0*	*1.0*

Since GWP estimation is commonly reported for 100 years' time horizon, short-term impacts were not evaluated within the scenarios of this work.

1.4. **Deviation range for UA-SA**

Table S8- Deviation range of inputs/outputs for SA and UA.

**Inputs/outputs**	**Deviation range**
Productivity (production line)	[0.90 - 1.10]
TCI	[0.60- 1.40]
OpEx	[0.60- 1.40]
Revenue (main)	[0.60- 1.40]
Carbon price for CBio	[0.50 - 1.50]
Power (renewable, wind)	[0.89 - 1.12]
Ethylene glycol	[0.77 - 1.29]
Sodium hydroxide	[0.77 - 1.29]
CO_2 utilisation	[0.77 - 1.29]
Water	[0.77 - 1.29]
Other auxiliary materials and wastes	[0.36 - 2.74]

The productivity in the factory was assumed to vary 10% in all productivity range set by LP and HP scenarios [$2.74 \times 10^{+7}$, $8.96 \times 10^{+7}$].

2. Result's data

In this section the results were reported as tables to facilitate access to the data.

The PCF was displayed in Table S9.

Table S9 - PCF of ethylene production.

Contribution analysis [kg of CO_{2eq}./ kg of the ethylene]	HP	LP
PCF, non-offset [h]	3.771	2.696
PCF[i]	1.628	0.332
Power	0.114	0.038
$Utilities_e$	0.910	0.206
$Utilities_w$	0.106	0.024
H_2O	0.001	0.006
Wastewater	0.277	0.975
NaOH	0.029	0.116
Bicarbonate	0.027	0.168
Glycerol	0.006	0.126
MeTHF	0.000	0.024
Ethanol	0.453	0.108
Ethylene glycol	0.257	0.528
CCU	-1.589	-0.414
Emissions (CO)	1.591	0.377
Wastewater treatment	-0.277	-0.975

[h] The emissions did not consider the offsetting of wastewater treatment or carbon capture and use.

[i] The emissions considered the treatment of wastewater with anaerobic digestion and carbon capture and use offsetting. All scenarios considered the BFG burden free.

The LCIA was displayed in Table S10.

Table S10- LCIA of ethylene production (normalised data).

Impact categories (EFv3.0)	Global normalisation factor [Unit/person][50]	Normalised data		
		LP	HP	Benchmark
PCF, non-offset	8.10E+03	3.33E-04	4.66E-04	1.88E-04
PCF, offset CCU	8.10E+03	1.61E-04	2.35E-04	1.88E-04
ACP	5.56E+01	2.40E-04	5.17E-04	8.33E-05
FWP	4.27E+04	1.41E-03	3.11E-03	1.03E-05
FEP	1.61E+00	3.85E-04	7.60E-04	6.34E-06
MEP	1.95E+01	1.18E-04	3.22E-04	4.57E-05
TEP	1.77E+02	1.57E-04	4.55E-04	5.48E-05
HTPc	1.69E-05	1.11E+00	1.45E-02	3.76E-06
ODP	5.36E-02	7.43E-05	2.19E-06	5.27E-08
IRP	4.22E+03	4.20E-05	3.86E-05	3.20E-08
LUP	8.19E+05	1.42E-05	4.73E-05	1.64E-08
REPm	6.36E-02	1.21E-03	2.04E-03	6.20E-07
ODP	5.36E-02	7.43E-05	2.19E-06	6.49E-09
PMP	5.95E-04	1.55E-04	3.81E-04	9.48E-05
WUP	1.15E+04	1.14E-04	2.28E-04	3.93E-05

The LCIA was displayed in Table S11.

Table S11- LCIA of ethylene production (non-normalised data).

Categories	LP	HP
**ACP**	1.34E-02	2.87E-02
**PCF, net**	3.32E-01	1.63E+00
**PCF, non-offset**	2.70E+00	3.77E+00
**GWPb**	4.23E-03	5.09E-03
**GWPf**	1.42E+00	1.97E+00
**GWPlluc**	2.02E-03	4.15E-03
**FWP**	6.02E+01	1.33E+02
**FWPi**	1.10E+01	1.70E+01
**FWPm**	4.12E+01	1.13E+02
**FWPo**	8.02E+00	2.85E+00
**ER, n-r**	2.97E+01	2.79E+01
**FEP**	6.19E-04	1.22E-03
**MEP**	2.30E-03	6.27E-03
**TEP**	2.78E-02	8.06E-02
**HTPc**	1.87E-05	2.44E-07
**HTPc,m**	1.30E-09	3.73E-09
**HTPc,o**	1.87E-05	2.41E-07
**HTPnc**	3.07E-07	1.22E-06
**HTPnc,m**	3.89E-08	1.12E-07
**HTPnc,o**	1.87E-05	2.41E-07
**IRP**	1.77E-01	1.62E-01
**LUP**	1.17E+01	3.87E+01
**REPm**	7.70E-05	1.30E-06
**ODP**	3.98E-06	1.17E-07
**PMP**	9.20E-08	2.27E-07
**WUP**	1.31E+00	2.62E+00

3. Graphical abstract

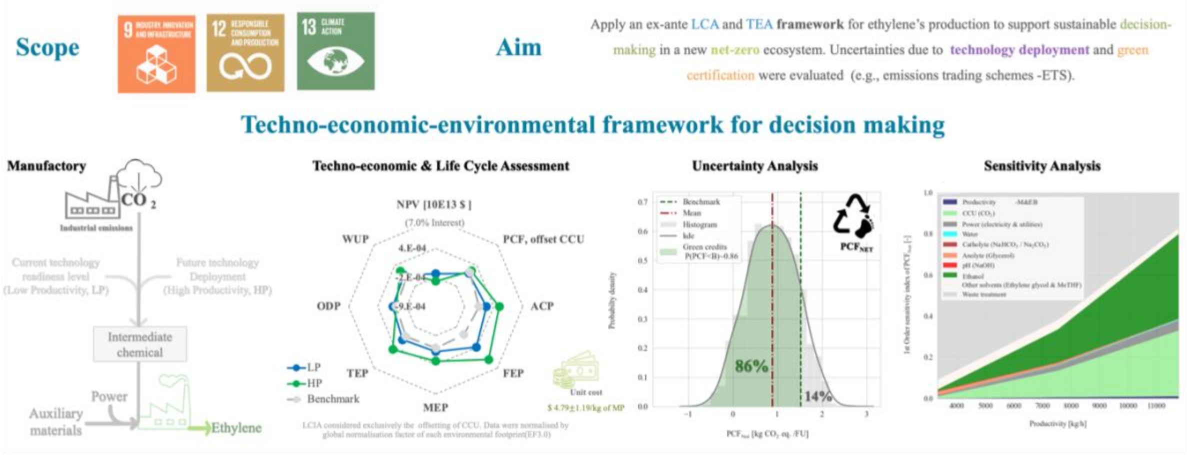

Figure S2- Graphical abstract.

REFERENCES

(1) Keep, M. ; J. I. ; W. M. *Contribution of the Steel Industry to the UK Economy*; UK, 2023. https://researchbriefings.files.parliament.uk/documents/CDP-2023-0016/CDP-2023-0016.pdf (accessed 2025-01-16).

(2) NREL. *Cost and Performance Baseline for Fossil Energy Plants Volume 1a: Bituminous Coal (PC) and Natural Gas to Electricity Revision 3*; 2015; Vol. 1a.

(3) Ogawa, T. Presumption on the Amount of Carbon and Hydrogen in Heavy Fuel Oils. *Journal of the Fuel Society of Japan* **1962**, *41* (1), 99–103.

(4) BEIS. *2018 Government GHG conversions Factors for Company Reporting. Department for Business, Energy and Industrial Strategy (BEIS) 2018:* 20-37. UK. https://assets.publishing.service.gov.uk/media/5b4f4703ed915d4397535e4e/2018_methodology_paper_FINAL_v01-00.pdf (accessed 2025-08-27).

(5) Conrado, R. J.; Gao, A. H. Integration of Fermentation and Gasification. US11097967B2, 2019. https://patents.google.com/patent/US11097967B2/en (accessed 2024-02-02).

(6) Van Den Bosch, B. ; F. M. C. ; Schouten, K. J. P. ; Brands, M. ; Rawls, B. J. ; Philips. M. F. Electrochemical Production of Formate. WO 2021/152054 A1, 2020.

(7) Cai, Y.; Wang, W.; Li, L.; Wang, Z.; Wang, S.; Ding, H.; Zhang, Z.; Sun, L.; Wang, W. Effective Capture of Carbon Dioxide Using Hydrated Sodium Carbonate Powders. *Materials* **2018**, *11* (2). https://doi.org/10.3390/ma11020183.

(8) Ye, X.; Lu, Y. Kinetics of CO2 Absorption into Uncatalyzed Potassium Carbonate-Bicarbonate Solutions: Effects of CO2 Loading and Ionic Strength in the Solutions. *Chem Eng Sci* **2014**, *116*. https://doi.org/10.1016/j.ces.2014.05.050.

(9) Li, T.; Shao, M. A Minireview on Electrochemical CO_2 Conversion Based on Carbonate/Bicarbonate Media. *EES Catalysis*. 2024. https://doi.org/10.1039/d3ey00287j.

(10) Knuutila, H.; Juliussen, O.; Svendsen, H. F. Kinetics of the Reaction of Carbon Dioxide with Aqueous Sodium and Potassium Carbonate Solutions. *Chem Eng Sci* **2010**, *65* (23). https://doi.org/10.1016/j.ces.2010.07.018.

(11) Park, K.; Lee, K. R.; Ahn, S.; Park, H.; Moon, S.; Yoon, S.; Jung, K. D. Investigating the Catalytic Deactivation of a Pd Catalyst during the Continuous Hydrogenation of CO2 into Formate Using a Trickle-Bed Reactor. *Catalysts* **2024**, *14* (3). https://doi.org/10.3390/catal14030187.

(12) Fernández-Caso, K.; Díaz-Sainz, G.; Alvarez-Guerra, M.; Irabien, A. Electroreduction of CO2: Advances in the Continuous Production of Formic Acid and Formate. *ACS Energy Letters*. 2023. https://doi.org/10.1021/acsenergylett.3c00489.

(13) Pittkowski, R.; Krtil, P.; Rossmeisl, J. Rationality in the New Oxygen Evolution Catalyst Development. *Current Opinion in Electrochemistry*. 2018. https://doi.org/10.1016/j.coelec.2018.11.014.

(14) Buchner, G. A.; Stepputat, K. J.; Zimmermann, A. W.; Schomäcker, R. Specifying Technology Readiness Levels for the Chemical Industry. *Industrial and Engineering Chemistry Research*. 2019. https://doi.org/10.1021/acs.iecr.8b05693.

(15) Pinto, A. S. S.; Gulpinar, N.; Liu, F.; Gibson, E.; Fuller, L.; Souter, P. Carbon Capture and Utilization for Sustainable Supply Chain Design of Intermediate Chemicals: The Formate Factory. *ACS Sustainable Resource Management* **2025**. https://doi.org/10.1021/acssusresmgt.4c00472.

(16) Ramdin, M.; Morrison, A. R. T.; De Groen, M.; Van Haperen, R.; De Kler, R.; Irtem, E.; Laitinen, A. T.; Van Den Broeke, L. J. P.; Breugelmans, T.; Trusler, J. P. M.; Jong,

W. De; Vlugt, T. J. H. High-Pressure Electrochemical Reduction of CO2 to Formic Acid/Formate: Effect of PH on the Downstream Separation Process and Economics. *Ind Eng Chem Res* **2019**, *58* (51). https://doi.org/10.1021/acs.iecr.9b03970.

(17) Jaimeferrer, J.; COUALLIER, E.; VIERS, P.; DURAND, G.; RAKIB, M. Three-Compartment Bipolar Membrane Electrodialysis for Splitting of Sodium Formate into Formic Acid and Sodium Hydroxide: Role of Diffusion of Molecular Acid. *J Memb Sci* **2008**, *325* (2), 528–536. https://doi.org/10.1016/j.memsci.2008.07.059.

(18) Luo, G. S.; Pan, S.; Liu, J. G. Use of the Electrodialysis Process to Concentrate a Formic Acid Solution. *Desalination* **2002**, *150* (3), 227–234. https://doi.org/10.1016/S0011-9164(02)00978-5.

(19) Laitinen, A. T.; Parsana, V. M.; Jauhiainen, O.; Huotari, M.; van den Broeke, L. J. P.; de Jong, W.; Vlugt, T. J. H.; Ramdin, M. Liquid–Liquid Extraction of Formic Acid with 2-Methyltetrahydrofuran: Experiments, Process Modeling, and Economics. *Ind Eng Chem Res* **2021**, *60* (15), 5588–5599. https://doi.org/10.1021/acs.iecr.1c00159.

(20) Cognion, J. M.; Durual, P. Process for the Manufacture of Ethylene from Ethyl Esters. US4620050A, 1984. https://patents.google.com/patent/US4620050A/en (accessed 2024-06-28).

(21) Lisnyanskii, I. M.; Zolotarev, N. S.; Sirotenko, A. A.; Buimov, A. A.; Gusev, V. D. Continuous Process for the Preparation of Ethyl Formate. *Pharm Chem J* **1969**, *3* (6). https://doi.org/10.1007/BF00764145.

(22) Menghai, W.. Semi-Continuous Producing Process for Ethyl Formate. CN1039226C, 1995. https://patents.google.com/patent/CN1039226C/en (accessed 2024-06-26).

(23) FDA. Part 184- Direct food substances affirmed as generally recognized as safe. Code of Federal Regulations by food and drug administration department of health and human services: USA. 2024.

(24) Yan, Z. ; D. Z. ; X. Y. ; L. W. ; J. G. ; S. Z. ; X. L. ; X. M. A Kind of Utilize Formic Acid and Ethanol Synthesis Ethyl Formate the Process of Refined Product, 2016.

(25) Wang, H.; Li, Y.; Su, W.; Zhang, Y.; Guo, J.; Li, C. Design and Control of Extractive Distillation Based on an Effective Relative Gain Array. *Chem Eng Technol* **2016**, *39* (12). https://doi.org/10.1002/ceat.201500202.

(26) Johnston, V. J. ; Z. J. H. ; C. J. T. ; C. L. ; K. B. F. Ethylene Production from Acetic Acid Utilizing Dual Reaction Zone Process. EP2318334A2, 2008.

(27) Balla, R.; Muthaiah, B.; Arathala, P. Experimental and RRKM Investigations on the Degradation of Ethyl Formate. *ChemistrySelect* **2017**, *2* (35). https://doi.org/10.1002/slct.201701927.

(28) Makens, R. F.; Eversole, W. G. Kinetics of the Thermal Decomposition of Ethyl Formate. *J Am Chem Soc* **1939**, *61* (11). https://doi.org/10.1021/ja01266a065.

(29) Cognion, J.-M. ; D. P. Process for the Preparation of Ethylene from Ethyl Esters. EP0176457A1, 1984. https://patents.google.com/patent/EP0176457A1/en (accessed 2025-06-23).

(30) Makens, R. F.; Eversole, W. G. Kinetics of the Thermal Decomposition of Ethyl Formate. *J Am Chem Soc* **1939**, *61* (11), 3203–3206. https://doi.org/10.1021/ja01266a065.

(31) Möller, T.; Filippi, M.; Brückner, S.; Ju, W.; Strasser, P. A CO 2 Electrolyzer Tandem Cell System for CO 2-CO Co-Feed Valorization in a Ni-N-C/Cu-Catalyzed Reaction Cascade. **2023**. https://doi.org/10.1038/s41467-023-41278-7.

(32) Green, W. B. ; G. V. E. Removal of Carbon Monoxide from Ethylene. US2973628A, 1958.

(33) Vuppaladadiyam, A. K.; Antunes, E.; Vuppaladadiyam, S. S. V.; Baig, Z. T.; Subiantoro, A.; Lei, G.; Leu, S. Y.; Sarmah, A. K.; Duan, H. Progress in the Development and Use of Refrigerants and Unintended Environmental Consequences. *Science of the Total Environment*. 2022. https://doi.org/10.1016/j.scitotenv.2022.153670.

(34) Li, R.; Ye, F.; Zhang, J.; Wang, M.; Li, K. Theoretical Analysis of Three CO2/C3H8 (R744-R290) Cascade Refrigeration Systems with Precooling Processes in Low-Temperature Circuits. *Appl Therm Eng* **2023**, *234*. https://doi.org/10.1016/j.applthermaleng.2023.121238.

(35) Barker, G. Towers. *The Engineer's Guide to Plant Layout and Piping Design for the Oil and Gas Industries* **2018**, 285–308. https://doi.org/10.1016/B978-0-12-814653-8.00011-4.

(36) Intratec. *Industrial Steam Cost | Industrial Utilities*; 2024. https://www.intratec.us/products/water-utility-costs/commodity/industrial-steam-cost (accessed 2024-05-20).

(37) Intratec. Industrial Steam Cost (Low Pressure) | Industrial Utilities. **2024**.

(38) Intratec. *Chilled Water Cost | Industrial Utilities*; 2024. https://www.intratec.us/products/water-utility-costs/commodity/chilled-water-cost (accessed 2024-05-20).

(39) Intratec. *Cooling Water Cost | Industrial Utilities*; 2024. https://www.intratec.us/products/water-utility-costs/commodity/cooling-water-cost (accessed 2024-05-20).

(40) EC. *EU Energy in Figures – Statistical Pocketbook 2023*; Publications Office of the European Union, 2023. https://doi.org/doi/10.2833/502436.

(41) Chemanalyst. *Ethylene Price Trend and Forecast*; 2024. https://www.chemanalyst.com/Pricing-data/ethylene-40 (accessed 2024-05-20).

(42) Intratec. *Glycerol Prices | Historical and Current*; 2024. https://www.intratec.us/chemical-markets/glycerol-price (accessed 2024-05-20).

(43) Chemanalyst. *Potassium Carbonate Price Trend and Forecast*; 2024. https://www.chemanalyst.com/Pricing-data/potassium-carbonate-1164 (accessed 2024-05-20).

(44) Indexbox. *Sodium Carbonate Market Analysis, Forecast Size, Trends and Insights.*; 2024. https://www.indexbox.io/blog/sodium-carbonate-price-per-ton-in-august-2022/#:~:text=In%20August%202022%2C%20the%20sodium%20carbonate%20price%20per,US%29%2C%20rising%20by%202.3%25%20against%20the%20previous%20month. (accessed 2024-05-20).

(45) PR. *Sodium Hydroxide Price Trend and Forecast*; 2022. https://www.procurementresource.com/resource-center/sodium-hydroxide-price-trends (accessed 2024-05-20).

(46) DESNZ. *Carbon Prices for Use in Civil Penalties*; UK Emissions Trading Scheme : UK, 2023. https://www.gov.uk/government/publications/determinations-of-the-uk-ets-carbon-price/uk-ets-carbon-prices-for-use-in-civil-penalties-2023 (accessed 2024-05-20).

(47) OCDE. *Effective Carbon Rates 2023*; OECD, 2023. https://doi.org/10.1787/b84d5b36-en.

(48) OCDE. *Carbon Pricing in the United Kingdom*; OCDE, 2021. www.oecd.org/tax/tax-policy/carbon-pricing-background-notes.pdf (accessed 2024-05-20).

(49) IPCC. *Working Group I: The Scientific Basis- Indirect GWPs*. https://archive.ipcc.ch/ipccreports/tar/wg1/249.htm#:~:text=Table%206.9%20shows%20that%20the,estimated%20at%202.8%20to%2010 (accessed 2025-06-23).

(50) Bassi, S. A.; Biganzoli, F.; Ferrara, N.; Amadei, A.; Valente, A.; Sala, S.; Ardente, F. Updated Characterisation and Normalisation Factors for the Environmental Footprint 3.1 Method. *Publications Office of the European Union* **2023**, *JRC130796*. https://doi.org/10.2760/798894.

LIST OF SUPLEMENTARY TABLES

Table S1- Mole fraction of fuels. 5

Table S2 - Boundaries for material, energy, and monetary flows used in 14

Table S3 - Physical properties and costs for utilities used in each scenario in SuperPro Process Design ®. 15

Table S4- Prices per unit of auxiliary inputs/outputs. 15

Table S5- Equipment summary. 16

Table S6- LCA's Inventory per functional unit (FU, 1 kg of ethylene). 17

Table S7- *Indirect GWP for CO in different time horizons* [49]. 18

Table S8- Deviation range of inputs/outputs for SA and UA. 18

Table S9 - PCF of ethylene production. 19

Table S10- LCIA of ethylene production (normalised data). 20

Table S11- LCIA of ethylene production (non-normalised data). 21

LIST OF SUPLEMENTARY FIGURES

Figure S1- Process diagram: LP(a) and HP(b). 13

Figure S2- Graphical abstract. 22
